# The Role of a Natural Amphibian Skin-Based Peptide, Ranatensin, in Pancreatic Cancers Expressing Dopamine D2 Receptors

**DOI:** 10.3390/cancers14225535

**Published:** 2022-11-10

**Authors:** Anna K. Laskowska, Mateusz Szudzik, Aneta Ścieżyńska, Michał Komorowski, Edina Szűcs, Dávid Gombos, Bartłomiej Bączek, Jowita Lipka-Miciuk, Sandor Benyhe, Patrycja Kleczkowska

**Affiliations:** 1 Department of Pharmaceutical Microbiology, Medical University of Warsaw, 02-106 Warsaw, Poland; 2 Department of Experimental Physiology and Pathophysiology, Centre for Preclinical Research (CBP), Medical University of Warsaw, 02-106 Warsaw, Poland; 3 Department of Histology and Embryology, Medical University of Warsaw, 02-106 Warsaw, Poland; 4 Institute of Biochemistry, Biological Research Centre of the Hungarian Academy of Sciences, Temesvári krt. 62, H-6726 Szeged, Hungary; 5 Doctoral School of Theoretical Medicine, Faculty of Medicine, University of Szeged, H-6726 Szeged, Hungary; 6 Department of Pharmacodynamics, Medical University of Warsaw, 02-106 Warsaw, Poland; 7 Military Institute of Hygiene and Epidemiology, 01-163 Warsaw, Poland; 8 Maria Sklodowska-Curie, Medical Academy in Warsaw, Solidarnosci 12 str., 03-411 Warsaw, Poland

**Keywords:** ranatensin, pancreatic cancer, anticancer agents, dopamine receptors

## Abstract

**Simple Summary:**

Cancer is one of the most problematic issues worldwide, as it still requires effective therapy. Unfortunately, clinically available cancer-specific medications result in serious side effects. This is also true for pancreatic cancer which additionally is well-known for difficulties in the treatment. Therefore, the aim of the study was to evaluate the biological activity, in terms of anticancer effects, of ranatensin (RAN), a naturally existing bombesin-like peptide. For the first time, we characterized the peptide as a dopaminergic system ligand. However, despite the presence of dopamine receptors in pancreatic cancer cell lines used, RAN was found to affect cancer cells possibly through different receptors since RAN’s impact on pancreatic cancer cells was not correlated with their expression level of DRD2. Nevertheless, this peptide may serve as a potential useful agent with therapeutic efficacy in cancers expressing DRD2 receptors, for which DRD2 antagonism is crucial to produce antitumor effects.

**Abstract:**

Despite the progress in early diagnostic and available treatments, pancreatic cancer remains one of the deadliest cancers. Therefore, there is an urgent need for novel anticancer agents with a good safety profile, particularly in terms of possible side-effects. Recently dopaminergic receptors have been widely studied as they were proven to play an important role in cancer progression. Although various synthetic compounds are known for their interactions with the dopaminergic system, peptides have recently made a great comeback. This is because peptides are relatively safe, easy to correct in terms of the improvement of their physicochemical and biological properties, and easy to predict. This paper aims to evaluate the anticancer activity of a naturally existing peptide—ranatensin, toward three different pancreatic cancer cell lines. Additionally, since there is no sufficient information confirming the exact character of the interaction between ranatensin and dopaminergic receptors, we provide, for the first time, binding properties of the compound to such receptors.

## 1. Introduction

Pancreatic cancer is one of the deadliest cancers with a poor prognosis and overall survival of 11% [1]. Indeed, despite developments in the detection and management of pancreatic cancer, the main problem is a late diagnosis and limited treatment options, as chemo- or radiotherapy are the only possibilities considered at later stages. Nevertheless, the golden standard for chemotherapy is gemcitabine, while other options include fluorouracil, FOLFIRINOX, erlotinib, nab-paclitaxel, and their combinations with other agents. Unfortunately, both the use and the efficacy of each agent depend on many factors and are fraught with side-effects [2,3]. Due to this, there is an urgent need for new treatment approaches. Recent studies have focused on immunotherapy or gene therapy. Furthermore, receptor-based targeted therapy has gained much attention. In fact, a great number of tumors express various neurotransmitter receptors [4,5], particularly dopamine receptors [6,7]. 

Dopamine (DA) and the dopaminergic system have been widely shown to be highly involved in cancer development and its treatment. There are five receptors DRD1, DRD2, DRD3, DRD4, and DRD5, which are divided into two subgroups: D1-like receptors (DRD1 and DRD5) and D2-like receptors (DRD2, DRD3, and DRD4) [8]. Among them, DRD1 and DRD2 subtypes are among the most studied in terms of their role in cancer growth and progression [9]. Several papers revealed increased mRNA and protein levels of DRD2 in pancreatic, colon, prostate, and breast cancers [10,11]. Similarly, DRD1 activation was suggested to be responsible for the enhanced antitumor activities in pancreatic cancer [12], although, in other tumor types, it behaves contradictorily (i.e., human hepatocellular carcinoma, breast cancer, etc.) [13,14]. This evidence suggests that changes in DA receptor expression mediated by the ligand may further alter diverse functions of cancer cells, indicating that modulation of the receptor activity greatly influences different types of cancer, as well as their biology and behavior. Moreover, it is known that a series of DA receptor-related ligands may be considered effective adjuvants to already used chemotherapy. This was true for DA, which positively affected the chemotherapeutic efficacy both in vitro and in vivo [15].

There are several compounds acting at dopamine receptors, either agonists or antagonists, which have already been on the market for many years. However, most of them are known for their usefulness in the treatment of mental disorders. Nevertheless, as with every drug, these were also found to induce clinically important undesirable side-effects, especially when possessing the ability to cross the blood–brain barrier. Therefore, since it is common to use naturally existing compounds as they reveal a better safety profile than their chemical analogues, much attention is given to endo- or exogenous peptides. Such an example is ranatensin (RAN), an undecapeptide (*p*Glu–Val–Pro–Gln–Trp–Ala–Val–Gly–His–Phe–Met.NH_2_) isolated from extracts of amphibian skin (northern leopard frog *Rana pipiens*) [16]. Although the literature is poor in information concerning RAN and its biological activity, there are suggestions for its possible activity in the area of DA-related oncological treatment. Indeed, in 1991 Zhu et al. demonstrated RAN to interact with DA receptors, as a DRD2 receptor antagonist, sulpiride, attenuated the RAN-induced antinociceptive effect in vivo [17]. Unfortunately, this paper remains the only one presenting the relationship between the DA system and RAN. Nonetheless, considering that RAN may act through DA receptors, it may influence DA receptor-expressing tumors. 

Therefore, the aim of this paper was to provide sufficient information on RAN interaction with the DA receptor system, particularly with DRD1 and DRD2, as well as determine its possible influence on three pancreatic carcinoma cell lines.

## 2. Materials and Methods

### 2.1. Drugs and Chemicals

Ranatensin (Cat. No. 4018117) was purchased from Bachem (Bubendorf, Switzerland). Dopamine (Cat. No H8502) and pimozide (Cat. No P1793) were obtained from Sigma-Aldrich (Poznan, Poland). Risperidone (Cat. No HY.B003) and gemcitabine (Cat. No HY.11013) were purchased from MedchemExpress (New Jersey, USA). Human DRD2/dopamine receptor D2 (Sandwich ELISA) ELISA Kit (LS-F4844) was obtained from LSBio (Seattle, USA). The CellTiter 96^®^ Aqueous One Solution Cell Proliferation Assay (MTS) (Cat. No G3581) was obtained from Promega (Walldorf, Germany). Polyclonal anti-beta actin antibody (Cat. No. ab8227) was obtained from Abcam (Cambridge, UK). DRD2 polyclonal antibody (Cat. No. PA5-115142), goat anti-rabbit IgG (H + L) secondary antibody, and HRP (Cat. No. 31460) were obtained from Invitrogen (Waltham, MA, USA).

Each binding assay reagent, including Tris-HCl, EGTA, NaCl, MgCl_2_, GDP, and GTPγS, was purchased from Sigma-Aldrich (Budapest, Hungary). The selective DRD1 antagonist, SCH-23390, and the selective DRD2 antagonist, spiperone, were obtained from Tocris Bioscience (Bristol, UK). The prepared ligand stock solution (1 mM) was stored at −20 °C. The radiolabeled GTP analogue, [^35^S]GTPγS, with a specific activity of 1000 Ci/mmol was obtained from Hartmann Analytic (Braunschweig, Germany). [^3^H]SCH-23390 (specific activity: 83.2 Ci/mmol), [^3^H]spiperone (specific activity: 80.2 Ci/mmol), and the UltimaGold^TM^ MV aqueous scintillation cocktail were purchased from PerkinElmer (Boston, MA, USA).

### 2.2. Receptor Binding Assay Ex Vivo

#### 2.2.1. Animals

Wistar rats (both sexes) were used for membrane preparations for binding studies. A temperature-controlled room (21–24 °C) under a 12 h/12 h light/dark cycle was provided for animals. Each rat had free access to water and food during the entire experiment. All the experiments that included animal use were conducted in accordance with the European Communities Council Directives (2010/63/EU) and the Hungarian Act for the Protection of Animals in Research (XXVIII.tv. 32.§). According to the 3R rule, we minimized the total number of animals, but to a number that allows for the reliability of the statistical analysis.

#### 2.2.2. Preparation of Brain Samples for Binding Assays

Rats were decapitated, and their brains and spinal cords were quickly removed. The brains and spinal cords were prepared for membrane preparation according to our previous protocols [18,19]. Briefly, the brains and spinal cords were homogenized in freshly prepared ice-cold Tris-HCl buffer (50 mM, pH 7.4) and centrifuged at 18,000 rpm for 20 min (4 °C). The resulting supernatant was removed while the pellet was taken up in the original volume of Tris-HCl buffer. The homogenate was further incubated at 37 °C for 30 min and again centrifuged. The received pellet was consequently suspended in 50 mM Tris-HCl (pH 7.4) buffer and kept until analysis at −80 °C. 

For the ligand-stimulated [^35^S]GTPγS binding experiments, animal brains were homogenized with a Dounce in five volumes (*v*/*w*) of ice-cold TEM (Tris-HCl, EGTA, and MgCl_2_) and stored at a temperature of −80 °C. The total protein concentration of the membrane preparation was determined by Bradford Assay [20].

#### 2.2.3. Receptor Binding Assays

##### Functional [^35^S]GTPγS Binding Experiments

Ligand-stimulated [^35^S]GTPγS binding experiments were performed as previously described [21,22] with slight modifications. Briefly, the membrane homogenates were incubated at a temperature of 30 °C for 60 min in Tris-EGTA buffer (pH 7.4). The buffer composition was as follows: 50 mM Tris-HCl, 1 mM EGTA, 8 mM MgCl_2_, 100 mM NaCl, and contained 20 MBq/0.05 cm^3^ [^35^S]GTPγS (0.05 nM) with increasing concentrations (10^−10^–10^−5^ M) of unlabeled ligands. The experiments were performed in the presence of GDP (30 μM). Basal [^35^S]GTPγS activity (total binding) was measured in the absence of the compounds tested, whereas nonspecific binding was determined in the presence of unlabeled GTPγS (10 μM). Filtration and subsequent rinsing with 5 mL of ice-cold 50 mM Tris-HCl (pH 7.4) buffer completed the reaction. The radioactivity measurements were carried out using UltimaGold^TM^ MV aqueous scintillation cocktail with Packard Tricarb 2300TR liquid scintillation counter. The results presented are from three separate [^35^S]GTPγS binding experiments.

##### Competitive Binding Experiments

In DRD1 displacement, the Tris-HCl buffer (pH 7.4) contained 120 mM NaCl, 5 mM KCl, 1 mM MgCl_2_, 2 mM CaCl_2_, and 1 µM mianserin. In DRD2 displacement, the Tris-HCl buffer (pH 7.4) was a mixture of 5 mM KCl, 2 mM MgCl_2_, 2 mM CaCl_2_, and 1 µM ketanserin. The incubation of tissue membranes was performed in the presence of the unlabeled ligands in increasing concentrations (10^−10^–10^−5^ M) at 35 °C for 45 min with [^3^H]SCH-23390 and at 25 °C for 120 min with [^3^H]spiperone. Either total or nonspecific binding was determined in the presence and absence of unlabeled dopaminergic receptor antagonists, SCH-23390 (DRD1) and spiperone (DRD2). Termination of the reaction and measurements of the radioactivity were carried out as mentioned above. The experiment was performed in duplicate and repeated at least three times.

### 2.3. Hemolytic Activity In Vitro

Ranatensin hemolytic activity was measured using a modified Mazzarino et al. protocol [23]. Briefly, human red blood cells (RBCs) were obtained from a healthy volunteer and centrifuged at 2500 rpm for 10 min at 4 °C. After the removal of plasma, pellets were resuspended in PBS and again centrifuged (2500 rpm for 10 min). This was performed in triplicate. RBCs were diluted in PBS to obtain a 10% suspension of RBC. The 10% RBC suspension was used to prepare the 2% RBC suspension and 100% hemolysis control. The 2% suspension of RBCs was incubated with ranatensin (0–200 μM) in a 1:1 ratio for 1, 2, and 4 h at 37 °C. The samples were again centrifuged at 4500 rpm (5 min). Then, supernatant (at a volume of 100 μL) from each sample was transferred to a 96-well plate. Optical density (OD) was measured at a wavelength of 540 nm. The positive control (100% hemolysis) was a 10% red blood cell suspension in distilled water (ratio 1:9). The 1% RBC suspension in PBS served as a negative control (0% hemolysis). The peptide-induced hemolysis was calculated according to the formula below.

Hemolysis [%] = (A − Ab)/(A100% − A0%) × 100%,

where A is the absorbance of the sample incubated with peptide, Ab is the absorbance of the blank sample, A100% is the absorbance of the reference (100% hemolysis), and A0% is the absorbance of the 1% hematocrit incubated with PBS (0% hemolysis).

Hemolysis assay was conducted under the approval of the Bioethics Committee—Commission for the Supervision of Research on People and Animals at CSK MSWiA in Warsaw (no. 67/2017).

### 2.4. Cell Culture Preparation and In Vitro Experiments

Pancreatic carcinoma cell lines, PANC-1 (ACC 783), BxPC-3 (ACC 760), and Capan-1 (ACC 244), were obtained from DSMZ (Braunschweig, Germany). Normal human fibroblasts (NHF; 106-05A) were purchased from Sigma-Aldrich (Poznan, Poland). PANC-1 and NHF cells were maintained in DMEM containing 10% fetal bovine serum (FBS) and 1% antibiotic solution. BxPC-3 and Capan-1 cells were maintained in RMPI-1460 with 15% FBS and 1% penicillin/streptomycin solution. Cells were regularly tested for mycoplasma contamination.

#### 2.4.1. Enzyme-Linked Immunosorbent Assay

Cells were seeded in six-well plates at a density of 3 × 10^5^ cells/well in duplicate and incubated up to 90% confluence. Cell lysates were prepared as per the manufacturer’s protocol. Briefly, cells were collected and centrifuged at 2500 rpm for 10 min. The supernatant was removed. Cells were washed three times with PBS and then resuspended in PBS. Cells were lysed by ultrasonication and centrifuged at 1500 rpm for 10 min at 4 °C and the supernatant was collected. The optical density of the samples was measured at 450 nm.

#### 2.4.2. Cell Viability

In all experiments, cells were seeded in 96-well plates at a density of 4 × 10^3^ (PANC-1, BxPC-3), 5 × 10^3^ (Capan-1), and 2 × 10^3^ (NHF) cells per well. Plates were incubated for 24 h at 37 °C.

To evaluate the effect of each compound on cell viability, cells were treated with different concentrations (0–60 µM) of tested compounds for 24, 48, and 72 h. Next, 20 µL of MTS solution was added to each well. Plates were incubated for 2 h at 37 °C. The absorbance was measured at 490 nm. Untreated cells served as a negative control. The number of viable cells is directly proportional to the intensity of color produced. Cell viability is expressed as a percentage of control cells.

To evaluate the activity of the compounds after blocking DRD2, cells were incubated with risperidone (10 µM) for 2 h at 37 °C. Next, the medium was removed and a fresh medium containing tested compounds (at a concentration of 0–60 µM) was added. Plates were incubated for a further 24 h. Next, 20 µL of MTS solution was added to each well, and incubation was started (for 2 h at 37 °C). The absorbance was measured at 490 nm. Untreated cells served as a negative control.

#### 2.4.3. Cell Migration (Scratch Assay)

In all experiments, cells were seeded in 24-well plates at a density of 7 × 10^3^ (PANC-1, BxPC-3) and 4 × 10^3^ (NHF) cells per well and incubated for 48 h. Next, clear lines were made using a sterile 10 μL pipette tip. The medium was carefully removed, and 500 µL of fresh medium was added to the cells. 

For evaluation of the effect of each compound on cell proliferation, cells were treated with different concentrations (0–40 µM) of tested compounds. Phase-contrast microscopic pictures were taken at 24, 48, and 72 h, and the area of the wound was measured using Image J software (National Institute of Health, V1.5, Bethesda, MA, USA). 

To evaluate the activity of the compounds after blocking DRD2, cells were incubated with risperidone (10 µM) for 2 h at 37 °C. Next, the medium was removed, and a fresh medium containing the tested compounds (0–60 µM) was added. Phase-contrast microscopic pictures were taken at 6 and 24 h. 

The cell migration rate was calculated by measuring the area of the wound at each timepoint. The measurements were performed using ImageJ software. The medium was not changed during the experiment. Due to its inability to form a confluent monolayer, cell migration was not evaluated for Capan-1 cells.

#### 2.4.4. Western Blot

Cells were seeded in T25 flasks at a density of 1 × 10^6^ cells per flask and incubated for 48 h at 37 °C. Next, cells were washed with PBS, collected using a cell scraper, and centrifuged (1500 rpm, 10 min, 4 °C). The supernatant was removed, and pellets were resuspended in 300 µL of RIPA buffer. Samples were incubated on ice for 30 min. Next, samples were homogenized and centrifuged (6000 rpm, 15 min, 4 °C). The supernatant was transferred to clean tubes, and protein concentration was determined using the Bradford protein assay. To determine the level of DRD2, all samples were resolved by electrophoresis using SDS-PAGE gels (10%). Electroblotting of the proteins on the PVDF membrane, their blocking with skimmed milk, and incubation with primary (1:1000) and secondary antibodies (1:10,000) were performed. Quantitative analysis of protein content was performed using a ChemiDoc MP Imaging System with Quantity One software (Bio-rad, Hercules, CA, USA).

#### 2.4.5. Statistical Analysis

For binding studies, experimental data were presented as the means ± standard error of the mean (SEM). All the results were demonstrated using GraphPad Prism 5.0 (GraphPad Prism Software Inc., San Diego, CA). The maximal stimulation (E_max_) of the receptors was presented as a percentage of the specific [^35^S]GTPγS binding observed over basal activity (settled as 100%). An unpaired t-test followed by a two-tailed p-value was used to determine the significance level. Significance was accepted at the *p* < 0.05 level.

In the competition binding assays, the IC_50_ value was calculated using ‘one site competition’ fitting. The inhibitory activity was presented as a percentage of the specific binding observed.

For in vitro experiments, the results were presented as the means ± SEM and analyzed using GraphPad Prism 5.0. To determine the statistical significance of the results, the normality of distribution followed by one-way ANOVA or nonparametric Kruskal–Wallis test was employed. To compare different timepoints or different groups, two-way ANOVA was followed by Bonferroni’s multiple comparisons. A *p*-value < 0.05 was considered statistically significant. 

## 3. Results

### 3.1. Ranatensin Affinity and Potency to Dopamine DRD1 and DRD2 Receptors

**Figure 1:**
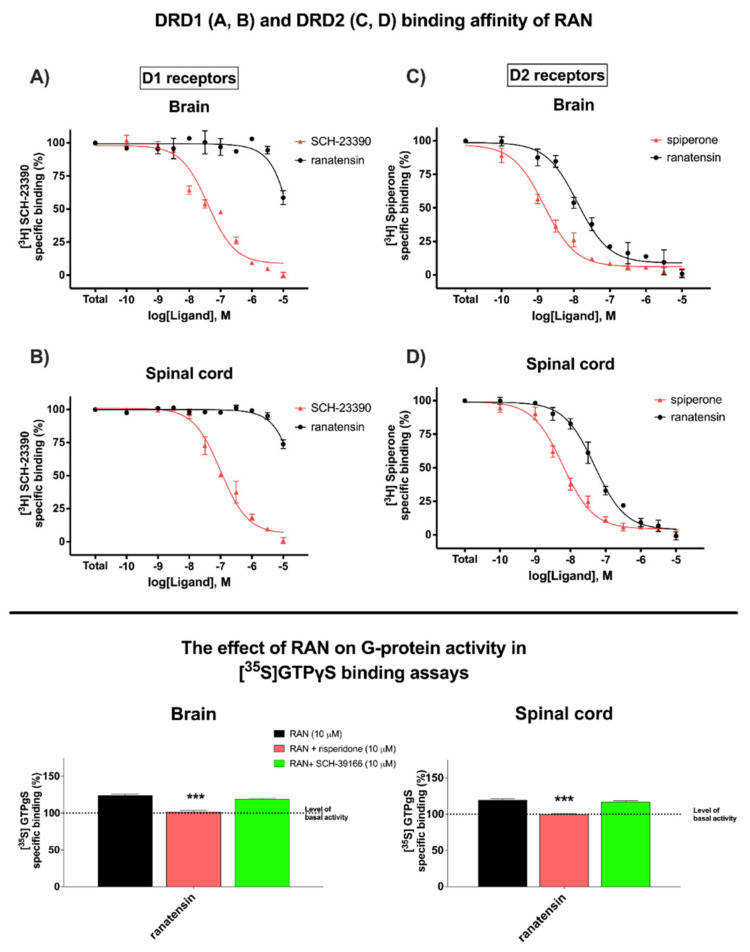
Binding affinities and G-protein activity in [^35^S]GTPγS binding assays of RAN. The top panel demonstrates DRD1 (**A**,**B**) and DRD2 (**C**,**D**) binding affinity of RAN compared to SCH−23390 and spiperone, respectively in [^3^H]SCH−23390 and [^3^H]spiperone competition binding assays in rat brain (**A**,**C**) and spinal cord (**B**,**D**) membrane homogenates. Membranes were incubated with 2 nM [^3^H]SCH−23390 and 0.25 nM [^3^H]spiperone. Values are presented as the means ± SEM for at least three experiments performed in duplicate. The bottom panel represents the intrinsic activity E_max_ of RAN in the absence or presence of the selective DRD1 antagonist SCH-39166 and the selective DRD2 antagonist risperidone in rat brain (left side) and spinal cord (right side) membrane homogenates. The level of basal activity was defined as 100%, and it is demonstrated with a dotted line. Points represent the means ± SEM for at least three experiments that were performed in triplicate. Statistical significances (*** *p* < 0.001) based on unpaired *t*-tests were noted for RAN vs. RAN + DRD2 antagonist.

Ranatensin (RAN) was examined in [^3^H]SCH-23390 and [^3^H]spiperone homolog displacement in rat brain and spinal cord homogenates. In the dopaminergic system RAN showed higher selectivity to DRD2 compared to DRD1 in rat brain membranes (DRD2: IC_50_ = 12.69 nM ± 1.21 vs. DRD1: IC_50_ > 10 000 nM) and spinal cord homogenates (DRD2: IC_50_ = 48.78 ± 1.15 vs. DRD1: IC_50_ > 10 000 nM) (Figure 1; top panel).

The maximal G-protein efficacy (E_max_) and potency (log EC_50_) of RAN in [^35^S]GTPγS binding assays in rat brain and spinal cord membrane homogenates were as follows: E_max_ = 124.1% ± 1.9% and log EC_50_ = −7.735 ± 0.224 M vs. E_max_ = 119.8% ± 1.3% and log EC_50_ = −6.108 ± 0.254 M. The selective DRD2 antagonist risperidone brought down the stimulation of RAN to basal activity in both the rat brain (E_max_ = 101.5% ± 1.7%; *p* < 0.001) and the spinal cords (E_max_ = 100.0% ± 0.7%; *p* < 0.001), respectively. On the other hand, the use of a selective DRD1 antagonist SCH-39166 did not cause a significant reversion of the efficacy of RAN in brain membranes (E_max_ = 119.1% ± 0.8%; *p* < 0.001) or in the spinal cord membranes (E_max_ was equal 117.4% ± 1.1%; *p* > 0.05) (Figure 1; bottom panel).

On the basis of the above results, further studies focused on DRD2 and RAN interactions.

### 3.2. Hemolytic Activity of Ranatensin in Terms of Its Potent Toxicity

**Figure 2:**
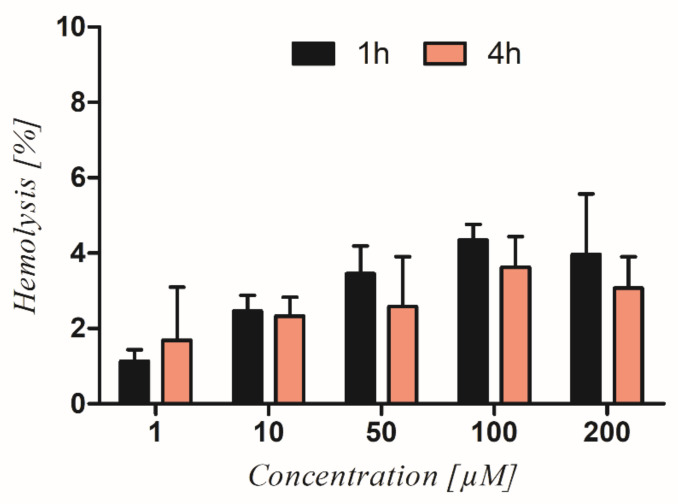
RAN-induced hemolysis. The graph shows the level of hemolysis of RBC treated with increasing concentrations of RAN after 1 and 4 h of treatment. Statistical analysis using two way-ANOVA followed by the Bonferroni correction revealed no significant differences (*p* > 0.05) between the results obtained.

The hemolytic activity of RAN was performed to preliminary determine its potential toxicity. The obtained results showed that the level of RAN-induced hemolysis was not time- (*p* = 0.521) or concentration-dependent (*p* = 0.193). The level of hemolysis after 1 and 4 h incubation with RAN ranged between 2% and 5%. There was no significant difference in RBC damage between 1 and 4 h of incubation with RAN (*p* > 0.05) (Figure 2). 

### 3.3. Dopamine D2 Receptors Expression in Pancreatic Cancer Cells 

**Figure 3:**
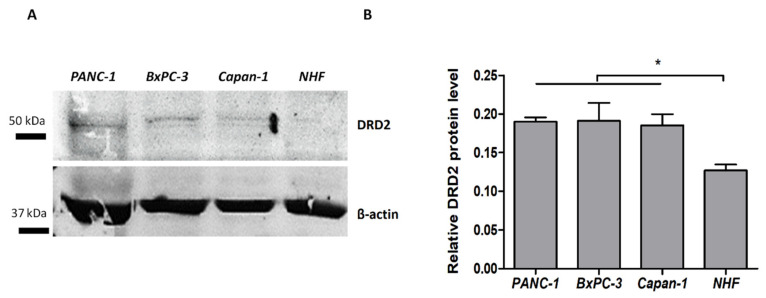
Representative Western blot images of DRD2 protein expression in cancer and normal cells (**A**). The quantitative analysis of the expression of DRD2 (mean ± SEM; expressed as a ratio to β-actin) shows different DRD2 expression levels in the tested cells (**B**). Statistical analysis was performed using one-way ANOVA with Tukey’s multiple comparison test. Results were considered statistically significant at * *p* < 0.05.

An enzyme-linked immunosorbent assay showed that all tested cell lines expressed DRD2 on their surface at different levels. The highest level of DRD2 expression was observed for BxPC-3, PANC-1, and Capan-1 cells, respectively. Normal fibroblasts (NHF) showed the lowest expression of DRD2. Western blot analysis also showed the highest DRD2 protein expression in BxPC-3, PANC-1, Capan-1 cells, and NHF, respectively (Figure 3A, Figure S1). The differences in expression levels between cancer cells were not statistically significant. The DRD2 protein level in fibroblasts was significantly lower compared to cancer cells (Figure 3B).

### 3.4. The Effect of Ranatensin on Pancreatic Cancer Cells 

**Figure 4:**
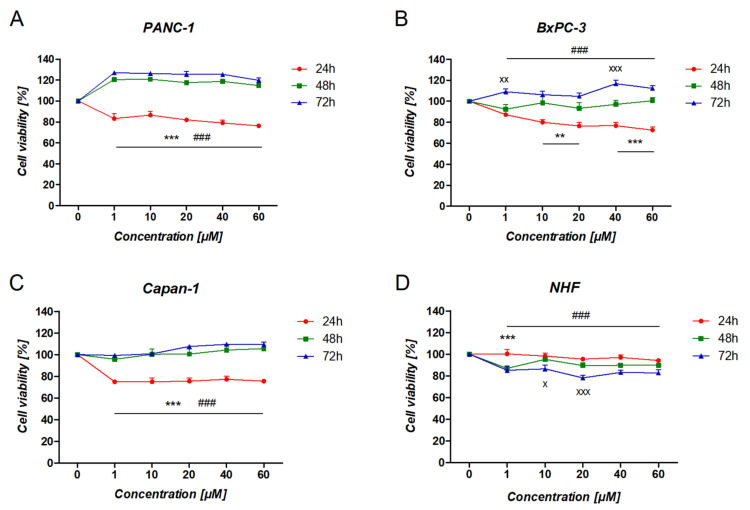
The viability of pancreatic cancer and normal cells treated with RAN. The graphs show the differential effect of RAN on tested cells at different timepoints. **A**–**D** represents various pancreatic cancer cell lines tested, i.e. (**A**) PANC-1; (**B**) BxPC-3; (**C**) Capan-1; and (**D**) NHF for normal human fibroblasts. For each line, differences in the viability of RAN-treated cells between three timepoints were compared. Statistical analysis performed using two-way ANOVA followed by Bonferroni post hoc test. Results were considered statistically significant as follows: ** *p* < 0.01, *** *p* < 0.005 for 24 h vs. 48 h; ^###^ *p* < 0.005 for 24 h vs. 72 h; ^x^ *p* < 0.05, ^xx^ *p* < 0.01, ^xxx^ *p* < 0.005 for 48 h vs. 72 h.

The effect of RAN on pancreatic cancer cells was time-dependent (*p* < 0.01) but not concentration-dependent (*p* > 0.05). As shown in Figure 4 (red line), cancer cells treated with RAN for 24 h showed lower viability compared to the untreated cells. However, the same effect was not observed after extended incubation (48 and 72 h) (Figure 4; blue and green lines). Prolonged incubation resulted in a significant increase in PANC-1 cell viability in comparison to the viability of the untreated cells (Figure 4A). A similar effect was observed for both BxPC-3 and Capan-1 cells (Figure 4B,C). Overall, the highest RAN activity was observed for BxPC-3, PANC-1, and Capan-1 cells, respectively. As for normal fibroblasts, RAN treatment for 24 h showed similar viability to untreated cells (Figure 4D red line). However, longer exposure of the cells to the action of RAN led to a reduction in fibroblast viability compared to untreated cells (Figure 4D; blue and green lines). 

All tested cell lines treated with pimozide (DRD2 antagonist) showed a significant decrease in cell viability after 24 h of exposure to the compound. PANC-1 cells were the most susceptible while fibroblasts were the most resistant (Table 1, Figure S2A). For cancer cells treated with risperidone (DRD2 antagonist) a decrease of up to 20% in cell viability was observed at the highest concentrations. There was no effect of risperidone on normal fibroblasts (Figure S2B). When treated with DA (DRD2 agonist), an increase in cell viability was observed for all tested cells (Figure S3A). The reference compound, gemcitabine, proved to be the most effective after 72 h of treatment (Table 1, Figure S3B). The BxPC-3 cells were the most susceptible (0.6426 ± 0.05 after 48 h; Table S1) while fibroblasts were the most resistant (Table 1).

**Figure 5:**
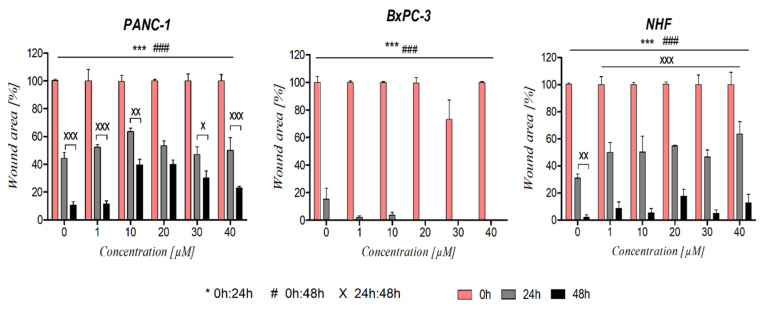
The influence of RAN on cell migration. Wound-healing assays were performed at 0, 24, and 48 h on RAN-treated and untreated PANC-1, BxPC-3, and NHF cells. For each cell line, differences in the migration of RAN-treated cells between three timepoints were compared. Wound area (%) was evaluated by the rate of cells migrating toward the center of the scratched area upon a time using ImageJ™ software. Images from a phase-contrast microscope showing changes in the area covered by the cells at 0, 24, and 48 h after wounding are presented in the Supplementary Materials in Figures S5–S7. Statistical analysis performed using two-way ANOVA followed by Bonferroni post hoc test. The obtained results were statistically significant as follows: *** *p* < 0.005 for 0 h vs. 24 h; ^###^ *p* < 0.005 for 0 h vs. 48 h; ^x^ *p* < 0.05, ^xx^ *p* < 0.01, ^xxx^ *p* < 0.005 for 24 h vs. 48 h, respectively.

Migration of RAN-treated PANC-1 and BxPC-3 cells was time- and concentration-dependent (*p* < 0.01). Fibroblast migration was only time-dependent. The highest migration rate was observed for BxPC-3 cells (Figure 5B). Total wound closure was observed after 24 h at a concentration ≥ 20 µM. The slowest wound closure rate was noted for PANC-1 cells (Figure 5A). 

In contrast to viability, DA did not significantly increase the cells’ ability to migrate. The effect of DA was not concentration-dependent (Figures S4A and S8). The effect of pimozide was concentration- and time-dependent. The migration rate of the cells decreased with the increasing concentration of pimozide (Figures S4B and S10). Migration of PANC-1 and NHF cells treated with risperidone was time- and concentration-dependent. For BxPC-3 cells, the migration rate was only time-dependent (Figures S4C and S9). When exposed to gemcitabine, all tested cells showed slower migration with increasing concentration of the compound (Figures S4D and S11). 

### 3.5. The Effect of DRD2 Blockage on Ranatensin Activity 

**Figure 6:**
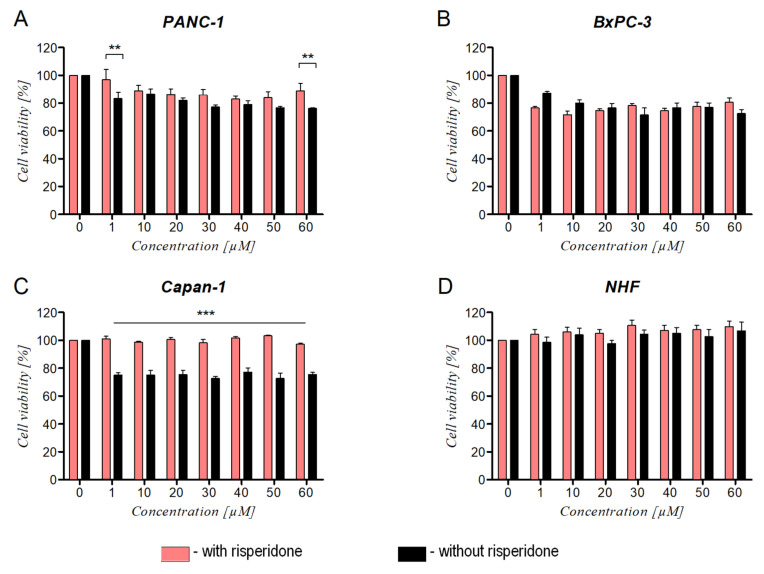
The effect of RAN on the viability of cells with and without risperidone-based inhibition of DRD2. **A**–**D** represents various pancreatic cancer cell lines, i.e. (**A**) PANC-1; (**B**) BxPC-3; (**C**) Capan-1; and (**D**) NHF for normal human fibroblasts. Cells with or without DRD2 blockade were treated with RAN for 24 h, and the differences in cell viability (%) were compared. Two-way ANOVA followed by Bonferroni correction was used to determine any statistical differences. Results were considered statistically significant as follows: ** *p* < 0.01, *** *p* < 0.005.

To determine whether RAN interacts with cells through DRD2, the cells were preincubated with risperidone and then treated with RAN (Figure 6) or other compounds (Figure S12). Blockade of DRD2 did not affect the activity of RAN on BxPC-3 and fibroblasts, as no statistically significant differences in cell viability were observed between cells preincubated with risperidone and those incubated only with RAN (Figure 6B,D). However, the previously observed viability-decreasing effect of RAN on Capan-1 and PANC-1 cells was not observed in risperidone-pretreated cells (Figure 6A,C). The loss of RAN activity against Capan-1 cells was significant. The viability of pretreated cells was similar to the viability of control cells, whereas the viability of RAN-only treated cells was decreased by around 20% compared to the control (Figure 6C). To a lesser extent, a loss of RAN activity against PANC-1 cells was observed (Figure 6A). Blocking the DRD2 localized on PANC-1 cells abolished the stimulating effect of DA. A similar effect was observed for BxPC-3 and NHF. The opposite effect was noted for Capan-1, cells but the differences between groups were not statistically significant (Figure S13A). Cells preincubated with risperidone were less sensitive to high concentrations of pimozide (≥ 30 µM; Table S2) than cells treated only with the drug. This effect was most noticeable for BxPC-3, NHF, and PANC-1 cells (Figure S13B). 

**Figure 7:**
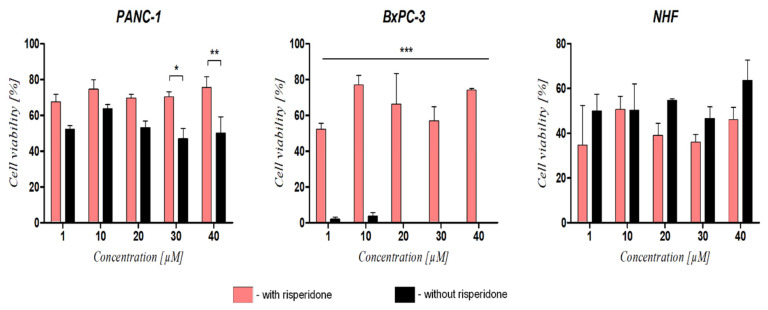
The effect of RAN on the migration of risperidone-pretreated cells and cells treated with RAN alone. Graphs present the comparison of migration rate between cells pretreated with risperidone and treated with RAN only for 24 h. Wound area (%) was evaluated as a function of the rate of cells migrating toward the center of the scratched area at a timepoint using ImageJ™ software. Statistical analysis was performed using two-way ANOVA with Bonferroni correction. For * *p* < 0.05, ** *p* < 0.01, and *** *p* < 0.005, results were considered statistically significant. Representative phase-contrast microscope images are presented in the Supplementary Materials in Figures S5–S7 and S14.

When exposed to RAN, the migration rate of DRD2-blocked PANC-1 and BxPC-3 cells was slower in comparison to cells treated with RAN alone. For BxPC-3 cells, the differences between groups were observed for all concentrations, whereas, for PANC-1, significant differences were noted for concentration values above 30 µM. For fibroblasts, differences between groups were noted but were not statistically significant (Figure 7). 

DRD2-blocked PANC-1 cells treated with pimozide or DA were shown to migrate much more slowly than cells with no risperidone pretreatment (Figures S17A,B and S18A,B). The differences were statistically significant (Figure S15). There were no statistically significant differences in the migration rate of DRD2-blocked BxPC-3 cells and cells treated with DA or pimozide solely. The opposite effect was observed for normal fibroblasts as risperidone-pretreated cells migrated more quickly than cells treated only with DA or pimozide (Figures S15, S17C and S18C). Since gemcitabine is known to affect cells through human equilibrative nucleoside transporter 1 receptors (hENT-1), it was excluded from this experiment.

## 4. Discussion

Since pancreatic cancer remains one of the most difficult types of tumors to treat with a poor prognosis of survival, many efforts were made to find an effective drug with an acceptable safety profile. In recent years, peptides have gained much attention as versatile tools for drug discovery, as well as delivery. These short-chain structures composed of amino acids possess various favorable properties, among which specific and high-affinity interactions with endogenous receptors are characteristic.

Ranatensin (RAN), a frog skin-derived undecapeptide highly homologous with bombesin, whose activity is attributed to binding to bombesin receptor subtypes was proposed to interact with dopamine receptors [17,24]. However, up to now, there has been no clear information on its binding with the abovementioned system. Our research not only confirmed this hypothesis but also revealed that RAN neither binds with high affinity nor activates the DRD1 receptor subtype, although DRD1 was slightly activated in rat brain membranes. In contrast, RAN showed high selectivity toward DRD2 compared to DRD1 and demonstrated its agonist behavior toward DRD2 (Figure 1). RAN is known as a bombesin-like peptide. Whereas both bombesin and bombesin-like peptides (i.e., neuromedin B, gastrin-releasing peptide GRP, etc.) target the GPCR receptors, i.e., the BB1 receptor being also a mammalian neuromedin B-preferring receptor (neuromedin B receptor), BB2 receptor (a GRP-preferring; GRP receptor), and an orphan receptor designated as bombesin receptor subtype-3 (BB3) [25], RAN, by interacting with a dopaminergic system, broadens the diversity of this group in terms of potential molecular targets.

This is important as the dopamine receptor system is strongly indicated to be involved in cancer development and progression. This is true for pancreatic cancer, for which the overexpression of dopamine receptors, particularly DRD2 [10], was found, although some findings suggest a greater role for DRD1 receptors, as it was established that DRD1 activation may enhance antitumor activities in pancreatic cancer [12]. While overexpression of DRD2 was associated with increased proliferation and tumor growth, its deactivation led to an antiproliferative effect [10]. This was confirmed in our research when cells were treated with a DRD2 agonist, dopamine, and its antagonists, pimozide or risperidone. We noted an increased proliferation of cancer cells treated with dopamine (Figure S4A) and the opposite effect when DRD2 antagonists were used (Figure S2). Intriguingly, however, our experiments showed that RAN may exert a different effect in terms of its potential antitumorigenic action. In fact, short-term incubation with RAN led to a decrease in the viability of BxPC-3, PANC-1, and Capan-1 cells (Figure 4A), thus demonstrating a behavior observed for DRD1 agonists. On the other hand, upon prolonging the time of incubation, an opposite effect was observed (Figure 4B). This may be attributed to protease activity and rapid RAN enzymatic degradation; however, additional research is needed. Considering the previously mentioned lack of relationship with DRD1, the reported beneficial effect noted after 24 h of incubation may be (at least partially) caused by the activity of the drug on other DA receptor subtypes, other G protein-coupled receptors (GPCRs), or other cellular targets. Liu et al. presented DRD4 activation to suppress the tumor-promoting inflammation of tumor-associated macrophages in pancreatic cancer [15]. Furthermore, pancreatic cancer was found rich in gastrin-releasing peptide receptors (GPR-Rs), and these were further described as an autocrine growth factor in various tumor cells [26,27]. Additionally, RAN had an equal affinity for GRP-Rs to GRP itself, although, in the literature, there is no information on its efficacy as an agonist, partial agonist, or antagonist [28]. Nonetheless, the exact mechanism indicating RAN’s pro- and antiproliferation effects remains unclear. 

Interestingly, the RAN cell viability suppressive effect did not correlate with the inhibition of cell migration as cells treated with RAN migrated at a similar rate to untreated cells (Figure 5). Another DRD2 agonist, dopamine, also did not significantly increase cancer cell motility (Figure S4A). Interestingly, inhibition of highly invasive PANC-1 cell migration was much stronger than for BxPC-3 cells (Figure 4A,B and Figure S4), although Stahle et al. demonstrated that PANC-1 cells had fivefold greater motility than BxPC-3 cells [29]. Various reports suggested that dopamine can increase or decrease cell proliferation and migration depending on the cancer’s tissue of origin. Huang et al. reported that treatment with dopamine, acting via DRD2, suppressed both the invasion and the migration of gastric cancer cells through the inhibition of the EGFR/AKT/MMP-13 pathway [30]. In lung cancer, overexpressed DRD2 or treatment with its agonist resulted in the inhibition of cancer progression [31,32]. However, many reports also indicate that excessive dopamine levels and upregulation of DRD2 can increase cell motility leading to metastasis [33,34,35]. It was shown that DRD2 antagonists such as pimozide or thioridazine inhibit both proliferation and migration of cancer cells, as well as induce apoptosis [10,36]. We also observed reduced motility of pancreatic cancer cells exposed to pimozide, as well as risperidone to a lesser extent (Figure S4).

To evaluate and confirm whether RAN affects cell behavior via DRD2, we pretreated cells with risperidone before exposing cells to RAN. In Capan-1 cells, the previously observed viability-decreasing effect of RAN was suppressed by the pretreatment with risperidone, which may indicate interactions via DRD2. However, DRD2 blockage affected RAN activity against PANC-1 and BxPC-3 cells to a much lesser extent as the differences were not statistically significant. However, risperidone pretreatment altered cancer cell migration. The proliferation-inducing effect of dopamine was suppressed in cells pretreated with risperidone. Double blockage with pimozide and risperidone did not significantly inhibit cell proliferation (Figure S12), although it inhibited migration of PANC-1 cells (Figures S14 and S15). The differences in cell susceptibility to RAN and other compounds may depend on the cell line and expression level of DRD2. Jandaghi et al. showed that the response to the DRD2 inhibitor differed according to pancreatic cell line depending on the expression level of DRD2 [10]. The abovementioned observations suggest that RAN may affect cancer cells through different receptors as RAN’s impact on pancreatic cancer cells was not correlated with their expression level of DRD2.

Another important aspect is RAN toxicity. The assessment of toxicity is a crucial step in the discovery of novel therapeutic agents, either natural or synthetic. Hemolytic activity is commonly used and may serve as a useful parameter demonstrating the level of possible compound-induced cytotoxicity [37,38]. In this aspect, our study demonstrated that RAN-induced hemolysis of red blood cells was low and did not exceed 5%. Additionally, we did not observe any significant differences between the concentration of RAN used or exposure time (Figure 2). Although these are preliminary studies, it seems that the results obtained speak in favor of the compound. Some anticancer peptides or peptides with dual antimicrobial–anticancer activity were shown to induce significant hemolysis, which hinders their clinical use [39,40].

## 5. Conclusions

The results of the experiments led us to indicate that RAN serves as a DRD2 agonist, although the exact mechanism of its action needs to be revealed. Therefore, this peptide may not be useful as a potent drug with therapeutic efficacy in cancers expressing DRD2 receptors, for which DRD2 antagonism is important to produce antitumor effects. Nonetheless, since RAN showed affinity for GRP-Rs, it might be important to determine whether RAN influences cell proliferation and migration via these receptors. Further studies are needed to fully evaluate and characterize RAN’s biological properties and its possible therapeutic use.

## Figures and Tables

**Table 1:** IC_50_ values for tested compounds after 24 and 72 h of treatment (µM).

	IC_50_ (µM)							
Cell Line	BxPC-3		Capan-1		PANC-1		NHF	
	24 h	72 h	24 h	72 h	24 h	72 h	24 h	72 h
**Ranatensin**	>60	>60	>60	>60	>60	>60	>60	>60
**Dopamine**	>60	>60	>60	>60	>60	>60	>60	>60
**Pimozide**	21.73 ± 2.27	17.8 ± 1.38	25.32 ± 3.11	19.96 ± 1.79	15.12 ± 1.51	20.34 ± 2.02	38.71 ± 1.37	23.57 ± 0.92
**Risperidone**	>60	>60	>60	>60	>60	>60	>60	>60
**Gemcitabine**	>60	0.2964 ± 0.15	>60	0.6566 ± 0.13	>60	0.2798 ± 0.031	>60	1.28 ± 0.1

## Data Availability

Upon request of those interested.

## Supplementary Materials

The following supporting information can be downloaded at: https://www.mdpi.com/article/10.3390/cancers14225535/s1, Figure S1: Uncropped Western blot of DRD2 protein expression in cancer and normal cells.; Figure S2: The viability of cells treated with dopamine D2 receptor antagonists, pimozide (**A**) and risperidone (**B**); Figure S3: The viability of cells treated with dopamine (DRD2 agonist) (**A**) and gemcitabine (**B**). The effect of each compound was evaluated in comparison to untreated cells (0 µM); Figure S4: The effect of compounds on cell migration; Figure S5: The effect of RAN on the migration of PANC-1 cells; Figure S6: The effect of RAN on the migration of BxPC-3 cells; Figure S7: The effect of RAN on the migration of normal fibroblasts; Figure S8: The effect of dopamine on the migration of cancer cells and normal fibroblasts; Figure S9: The effect of risperidone on the migration of cancer cells and normal fibroblasts; Figure S10: The effect of pimozide on the migration of cancer cells and normal fibroblasts; Figure S11: The effect of gemcitabine on the migration of cancer cells and normal fibroblasts; Figure S12: The effect of DRD2 agonists (**A**,**B**) and antagonist (**C**) on the viability of risperidone-pretreated cells after 24 h incubation; Figure S13: The comparison between the viability of risperidone-pretreated cells exposed to dopamine (**A**) or pimozide (**B**) and cells treated with dopamine or pimozide alone for 24 h; Figure S14: The effect of compounds on the cell migration of risperidone-pretreated cells; Figure S15: Comparison of the effect of dopamine and pimozide on the migration of risperidone-pretreated cells and cells treated with dopamine and pimozide alone after 24 h; Figure S16: The effect of ranatensin on the cell migration of risperidone-pretreated cells; Figure S17: The effect of dopamine on the cell migration of risperidone-pretreated cells; Figure S18: The effect of pimozide on the cell migration of risperidone-pretreated cells; Table S1: IC_50_ values (µM) of compounds after 48 h of treatment; Table S2: IC_50_ values of DRD2 -blocked cells treated with pimozide for 24 h (µM)
