# Supplementary material for: The Role of a Natural Amphibian Skin-Based Peptide, Ranatensin, in Pancreatic Cancers Expressing Dopamine D2 Receptors
Source: Cancers (Basel). 2022 Nov 10;14(22):5535. doi: 10.3390/cancers14225535 (PMC9688159; doi:10.3390/cancers14225535)
Supplement: Supplementary file 1 [file cancers-14-05535-s001.zip › cancers-1976959-supplementary.pdf]

Supplementary materials

The role of natural amphibian skin-based peptide, ranatensin, in pancreatic cancer expressing dopamine D2 receptors

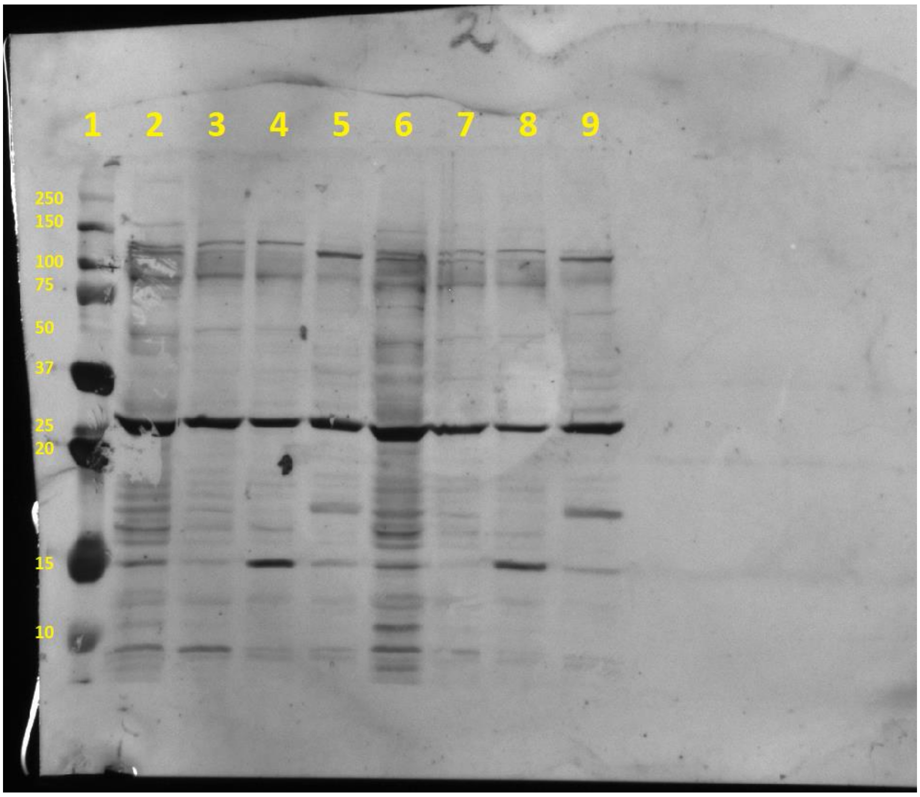

Figure S1. Uncropped Western blot of DRD2 protein expression in cancer and normal cells.
Line 1: Precision Plus Protein Dual Color Standard (Bio-Rad, Hercules, CA, USA); Lines 2-5 represent DRD2 expression in PANC-1 (2), BxPC-3 (3), Capan-1 (4), and NHF (5), and were also presented in Figure 3 in the main text. Lines 6-9 represent the second repetition of tested samples: PANC-1 (6), BxPC-3 (7), Capan-1 (8), and NHF (9).

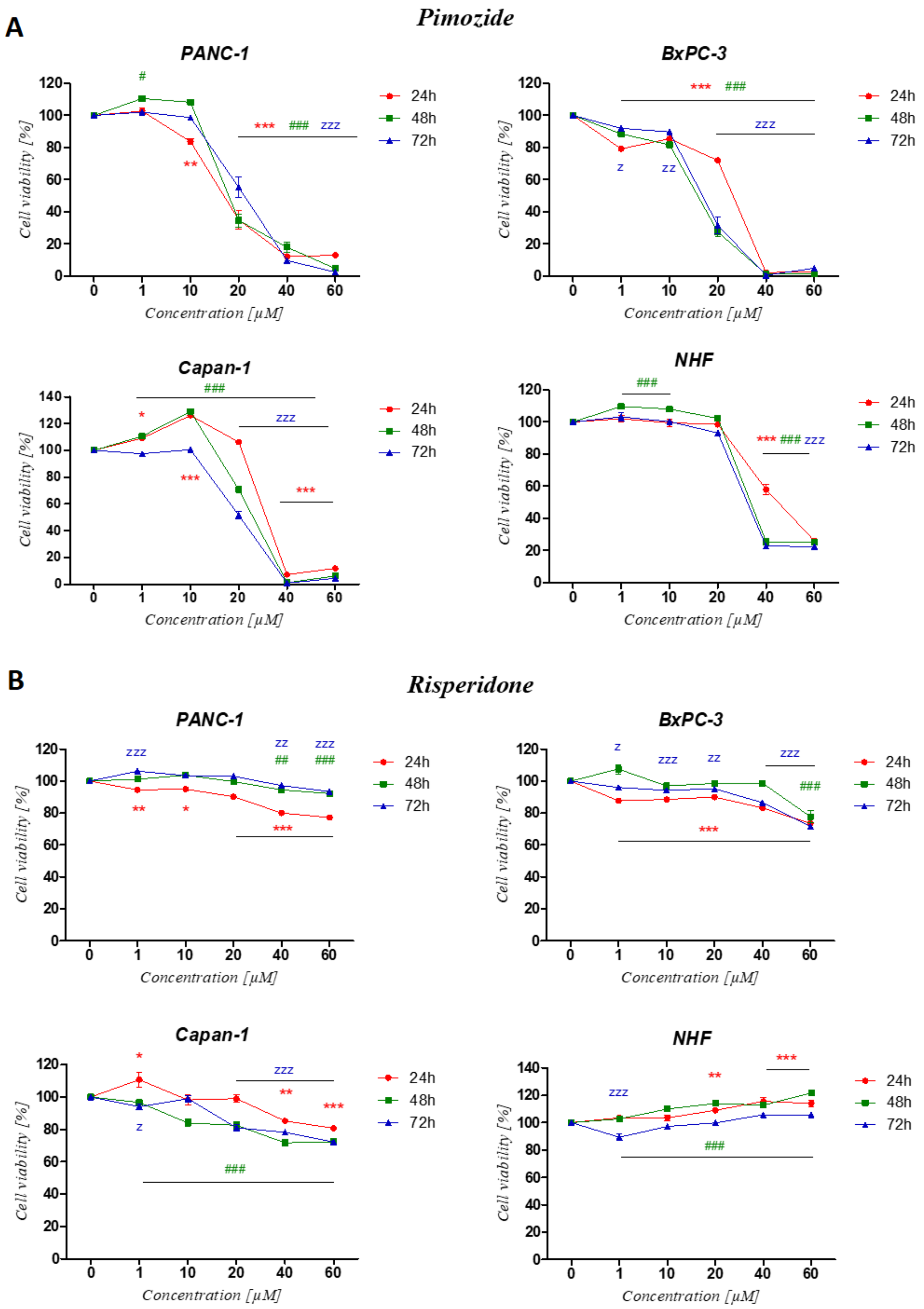

Figure S2. The viability of cells treated with dopamine D2 receptor antagonists, pimozide (A) and risperidone (B). The effect of each compound was evaluated in comparison to untreated cells (0 µM). Statistical analysis was performed using one-way ANOVA followed by Dunnett's multiple comparison test. Results were considered statistically significant when: *P < 0.05; **P < 0.01; ***P < 0.005., ^{z}P < 0.05; ^{zz}P < 0.01; ^{zzz}P < 0.005, as well as $^{\#\#}$P < 0.01; $^{\#\#\#}$P < 0.005

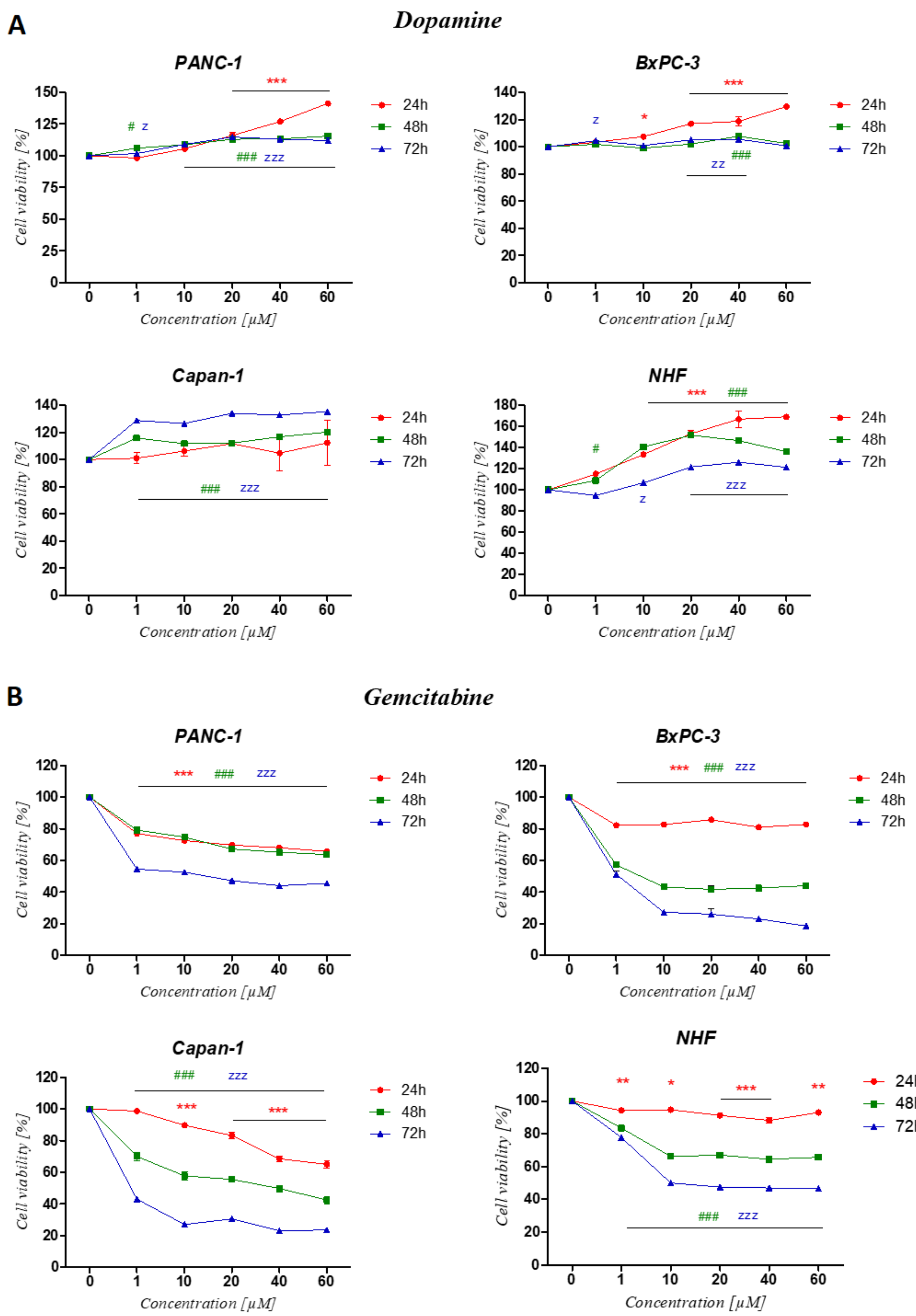

Figure S3. The viability of cells treated with dopamine (DRD2 agonist) (A) and gemcitabine (B). The effect of each compound was evaluated in comparison to untreated cells (0 µM). Statistical analysis was performed using one-way ANOVA followed by Dunnett's multiple comparison test. Results were considered statistically significant when: $^{*}P < 0.05$; $^{**}P < 0.01$; $^{***}P < 0.005$., $^{z}P < 0.05$; $^{zzz}P < 0.005$, as well as $^{###}P < 0.005$

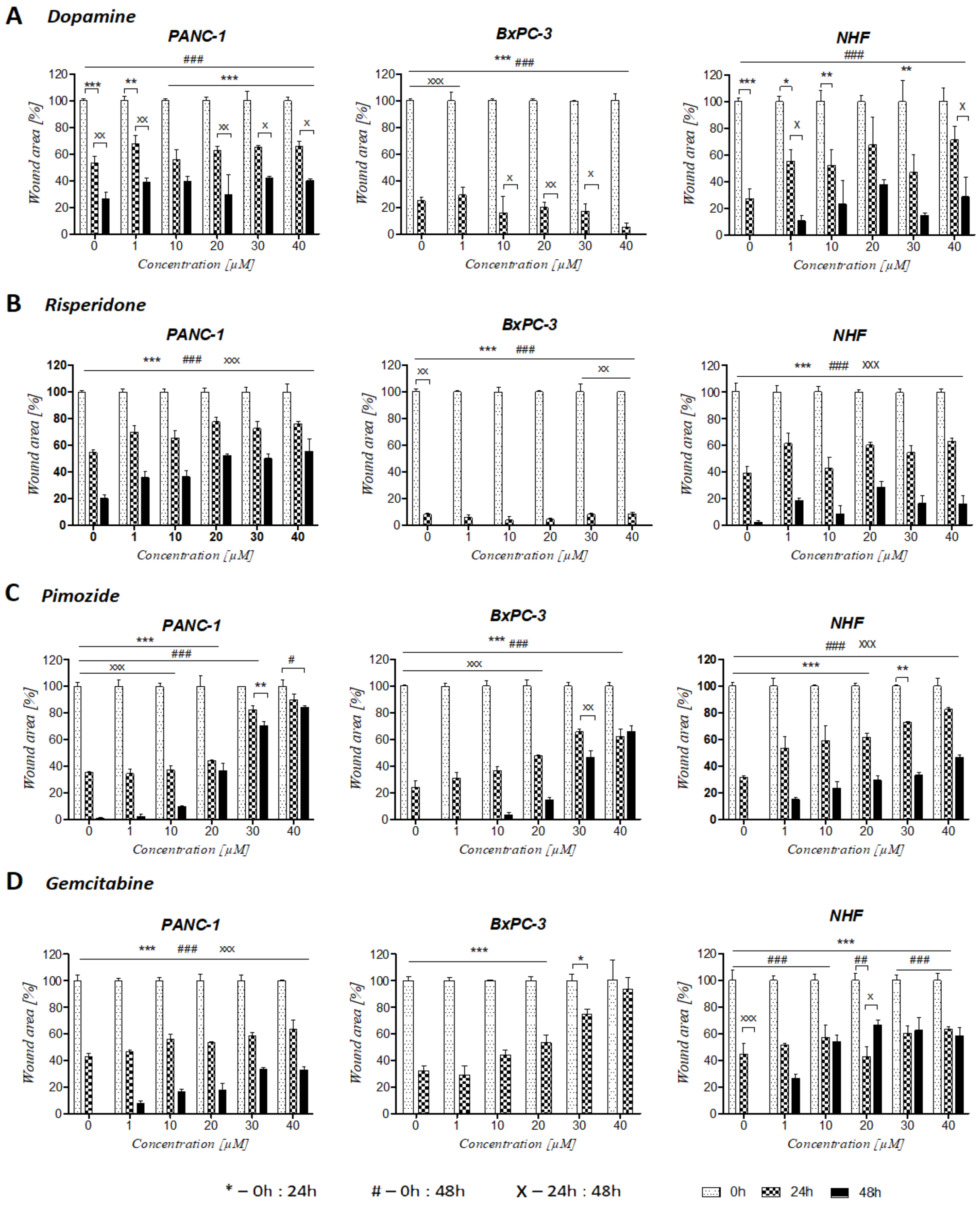

Figure S4. The effect of compounds on cell migration. Representative phase-contrast microscope images showing the area covered by the cells at 0, 24, and 48h after wounding, and presented in Figure S10. Original magnification 10x. Wound area (%) was determined by the rate of cells moving towards the scratched area upon time using ImageJ ™ software. Differences in wound area of cells treated with increasing concentrations of RAN at different time points were analyzed. Statistical analysis performed using two-way ANOVA followed by Bonferroni post-tests. Results were considered statistically

significant when: *P < 0.05; ** P < 0.01; ***P < 0.005 for 0 h vs 24 h, $^{\#}P < 0.05$; $^{\#\#}P < 0.01$; $^{\#\#\#}P < 0.005$ for 0 h vs 48 h, $^{x}P < 0.05$; $^{xx}P < 0.01$; $^{xxx}P < 0.005$ for 24 h vs 48 h.

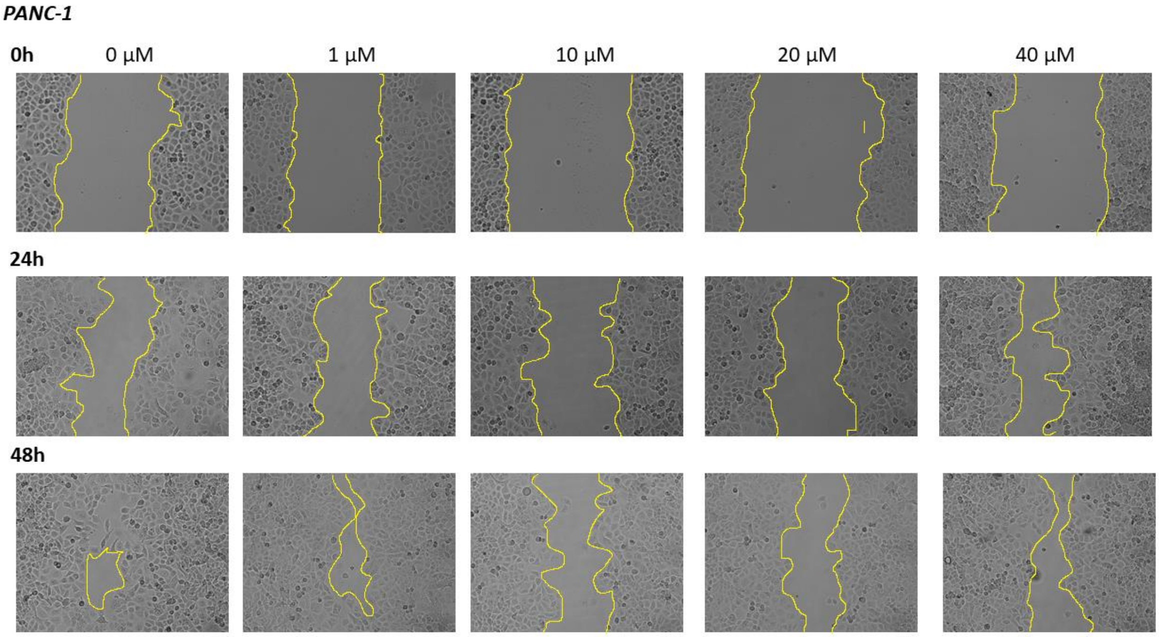

Figure S5. The effect of RAN on the migration of PANC-1 cells. Wound-healing assays were performed at 0, 24, and 48h on RAN-treated and untreated PANC-1 cells. Representative phase-contrast microscope images showing the area covered by the cells at 0, 24, and 48h after wounding. Original magnification 10x. Wound area (%) was determined by the rate of cells moving towards the scratched area upon time using ImageJ ™ software, and presented in Figure 5.

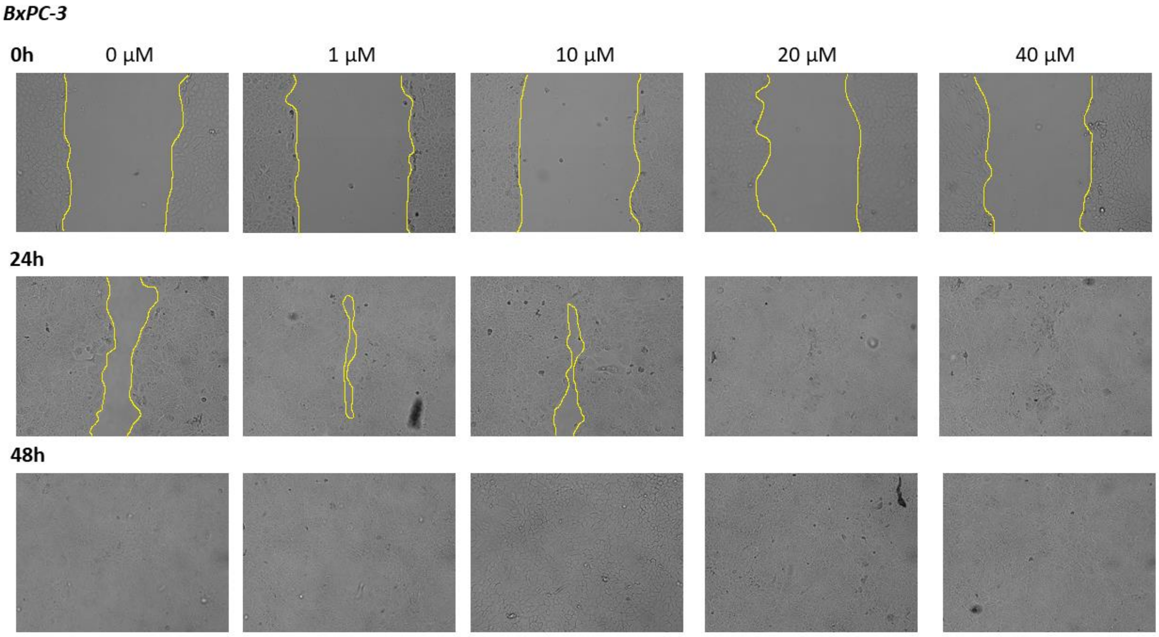

Figure S6. The effect of RAN on the migration of BxPC-3 cells. Wound-healing assays were performed at 0, 24, and 48h on RAN-treated and untreated BxPC-3 cells. Representative phase-contrast microscope images showing the area covered by the cells at 0, 24, and 48h after wounding. Original magnification

10x. Wound area (%) was determined by the rate of cells moving towards the scratched area upon time using ImageJ ™ software, and presented in Figure 5.

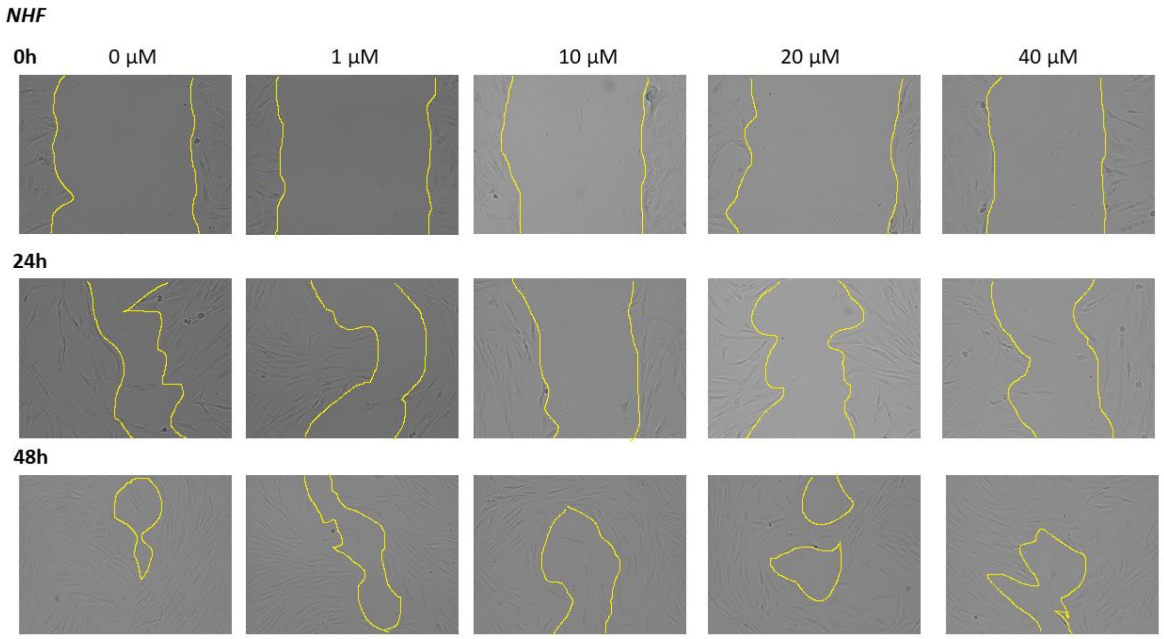

Figure S7. The effect of RAN on the migration of normal fibroblasts. Wound-healing assays were performed at 0, 24, and 48h on RAN-treated and untreated fibroblasts. Representative phase-contrast microscope images showing the area covered by the cells at 0, 24, and 48h after wounding. Original magnification 10x. Wound area (%) was determined by the rate of cells moving towards the scratched area upon time using ImageJ ™ software, and presented in Figure 5.

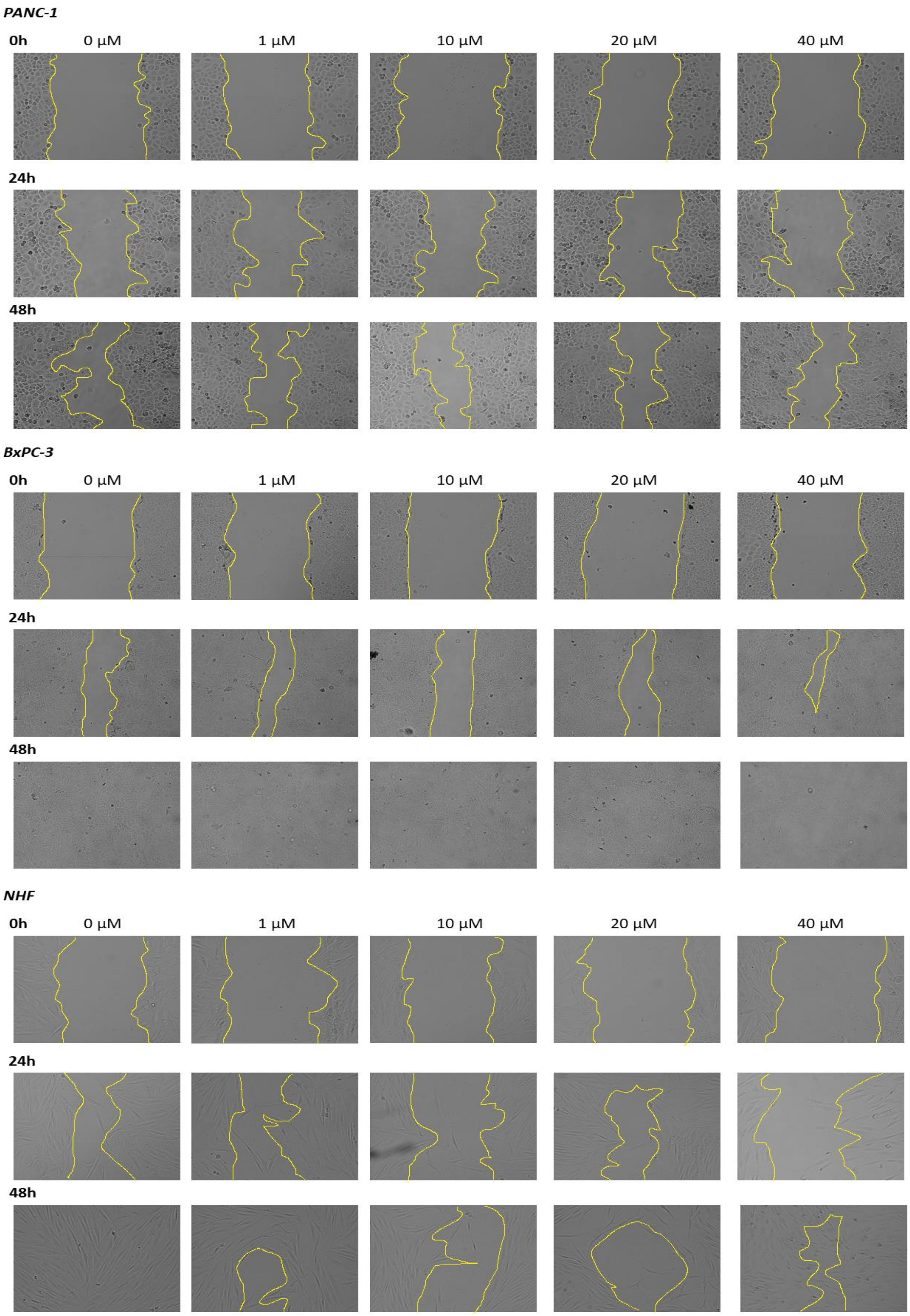

Figure S8. The effect of dopamine on the migration of cancer cells and normal fibroblasts. Wound-healing assays were performed at 0, 24, and 48h on dopamine-treated and untreated cells. Representative phase-contrast microscope images showing the area covered by the cells at 0, 24, and 48h after wounding. Original magnification 10x. Wound area (%) was determined by the rate of cells moving towards the scratched area upon time using ImageJ ™ software, and presented in Figure S4A.

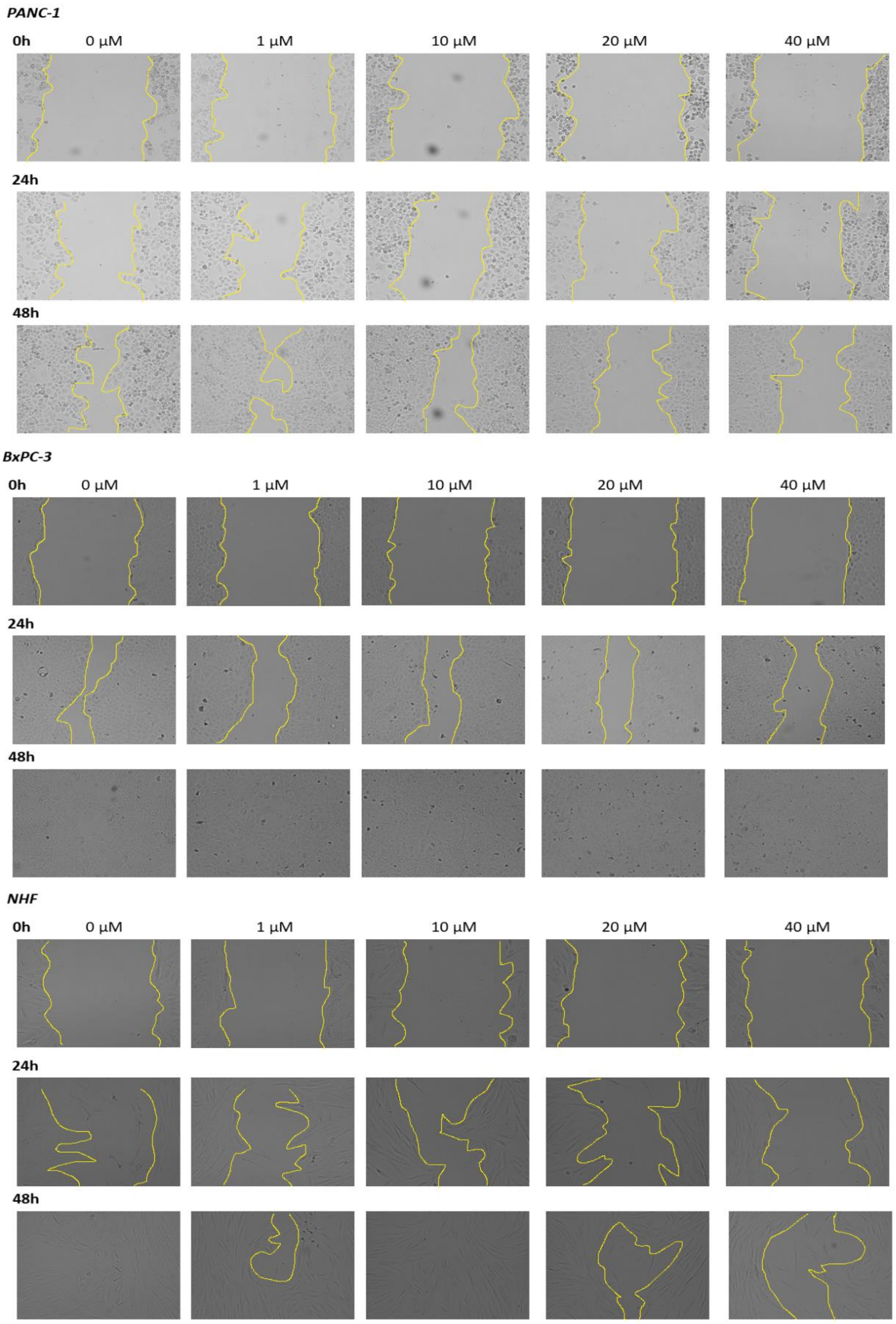

Figure S9. The effect of risperidone on the migration of cancer cells and normal fibroblasts. Wound-healing assays were performed at 0, 24, and 48h on risperidone-treated and untreated cells. Representative phase-contrast microscope images showing the area covered by the cells at 0, 24, and 48h after wounding. Original magnification 10x. Wound area (%) was determined by the rate of cells moving towards the scratched area upon time using ImageJ ™ software, and presented in Figure S4B.

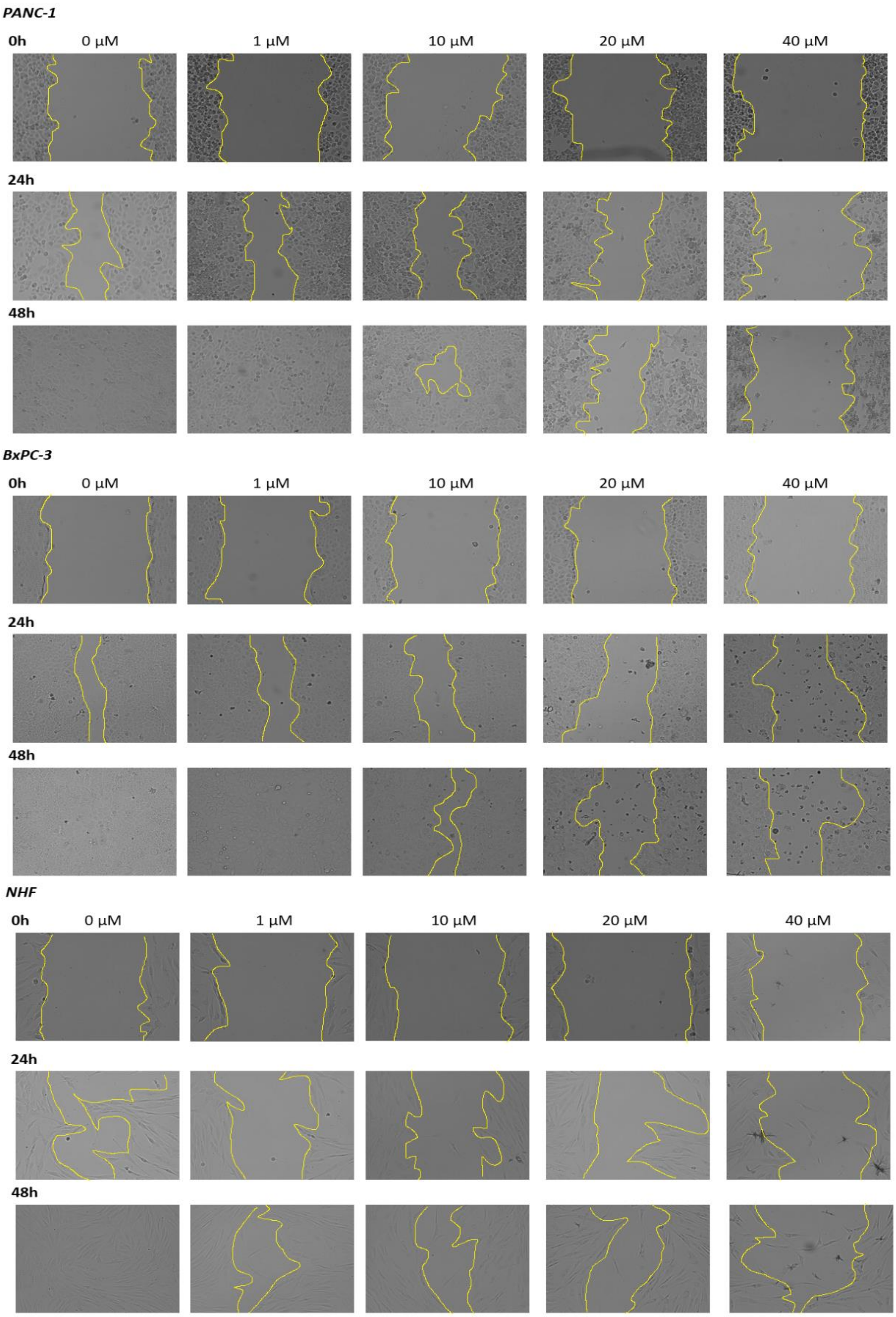

Figure S10. The effect of pimozide on the migration of cancer cells and normal fibroblasts. Wound-healing assays were performed at 0, 24, and 48h on pimozide-treated and untreated cells. Representative phase-contrast microscope images showing the area covered by the cells at 0, 24, and 48h after wounding. Original magnification 10x. Wound area (%) was determined by the rate of cells moving towards the scratched area upon time using ImageJ ™ software, and presented in Figure S4C.

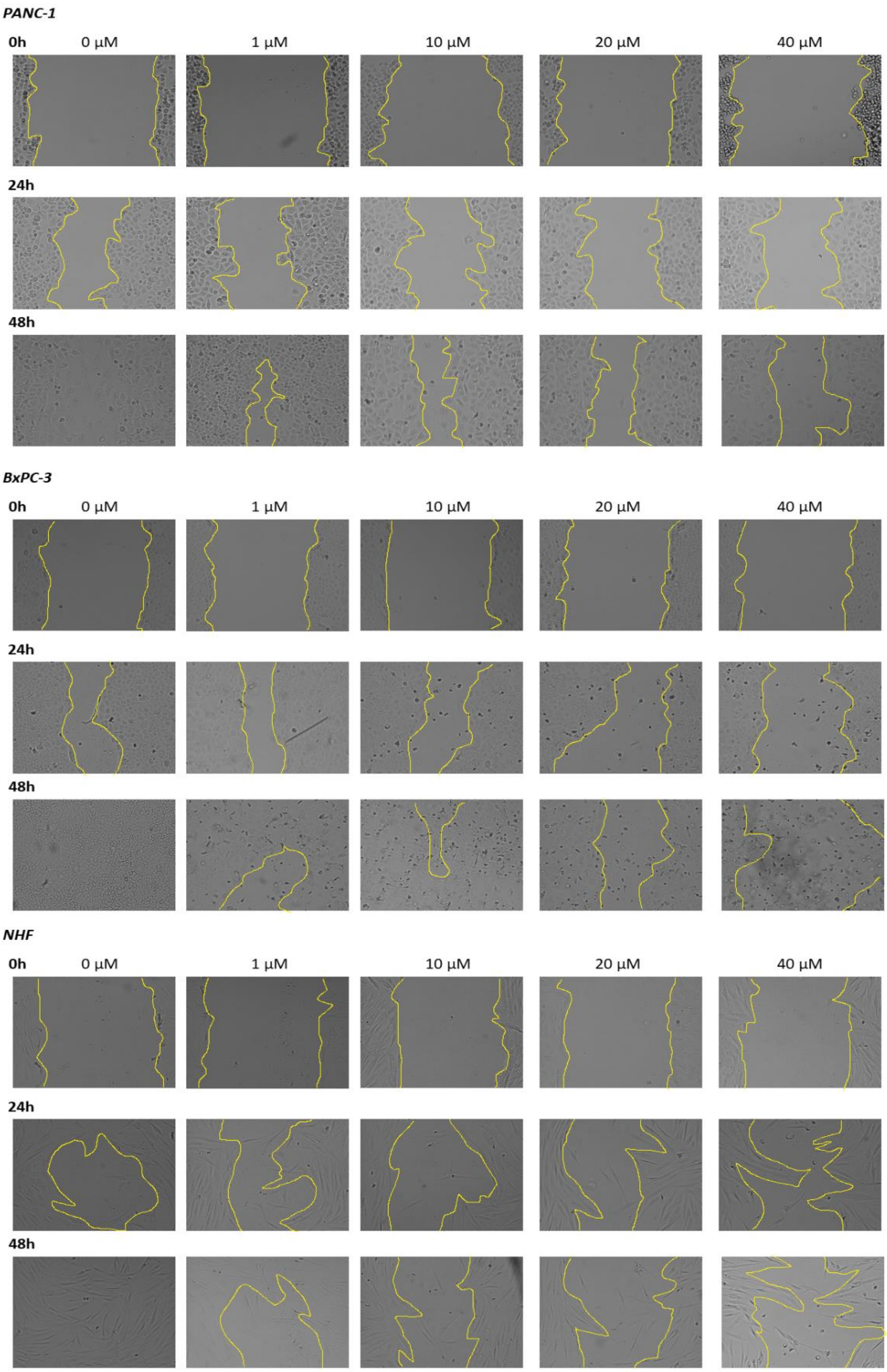

Figure S11. The effect of gemcitabine on the migration of cancer cells and normal fibroblasts. Wound-healing assays were performed at 0, 24, and 48h on gemcitabine-treated and untreated cells. Representative phase-contrast microscope images showing the area covered by the cells at 0, 24, and 48h after wounding. Original magnification 10x. Wound area (%) was determined by the rate of cells moving towards the scratched area upon time using ImageJ ™ software, and presented in Figure S4D.

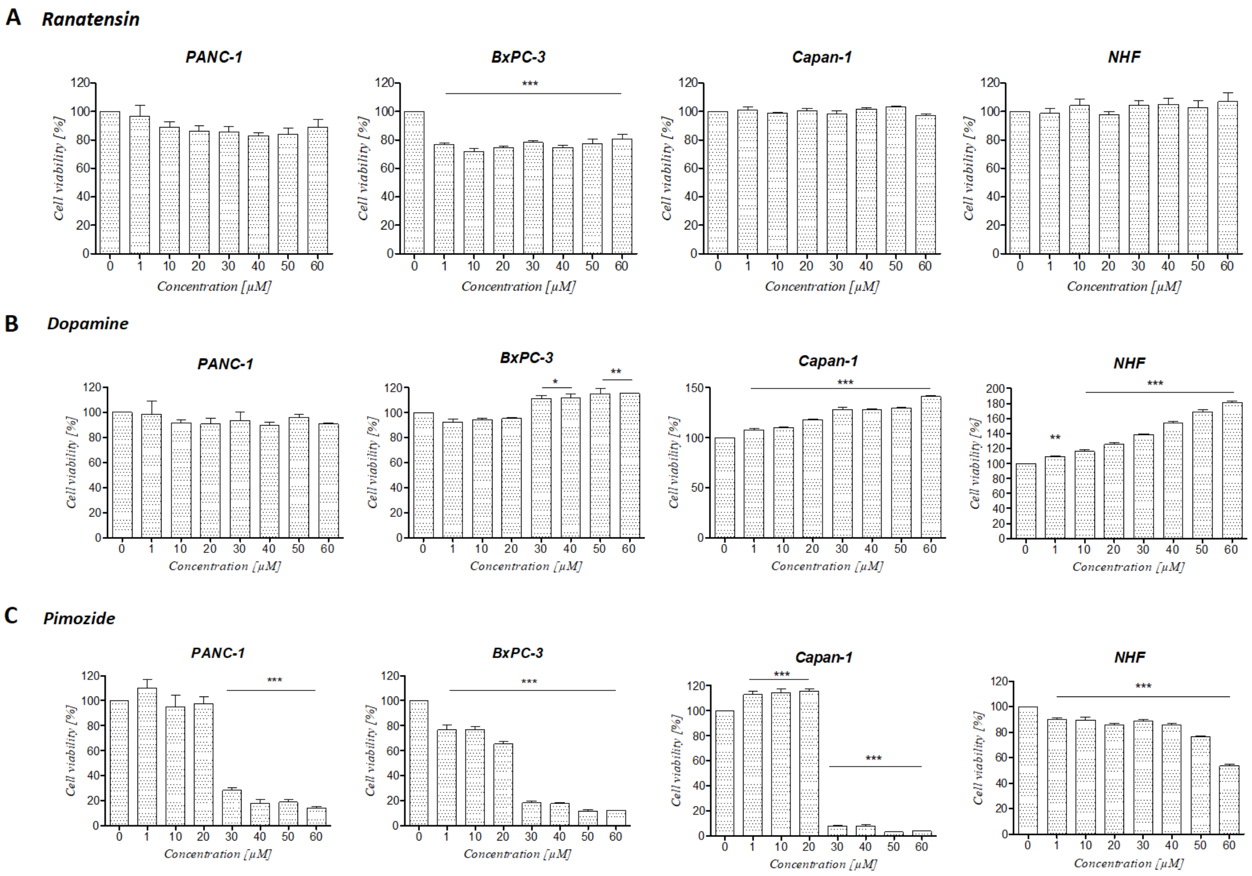

Figure S12. The effect of DRD2 agonists (A, B) and antagonist (C) on the viability of risperidone-pretreated cells after 24 h incubation. Statistical analysis was performed using one way ANOVA followed by Dunnett's multiple comparison test. Results were considered statistically significant when: *$P < 0.05$; **$P < 0.01$; ***$P < 0.005$.

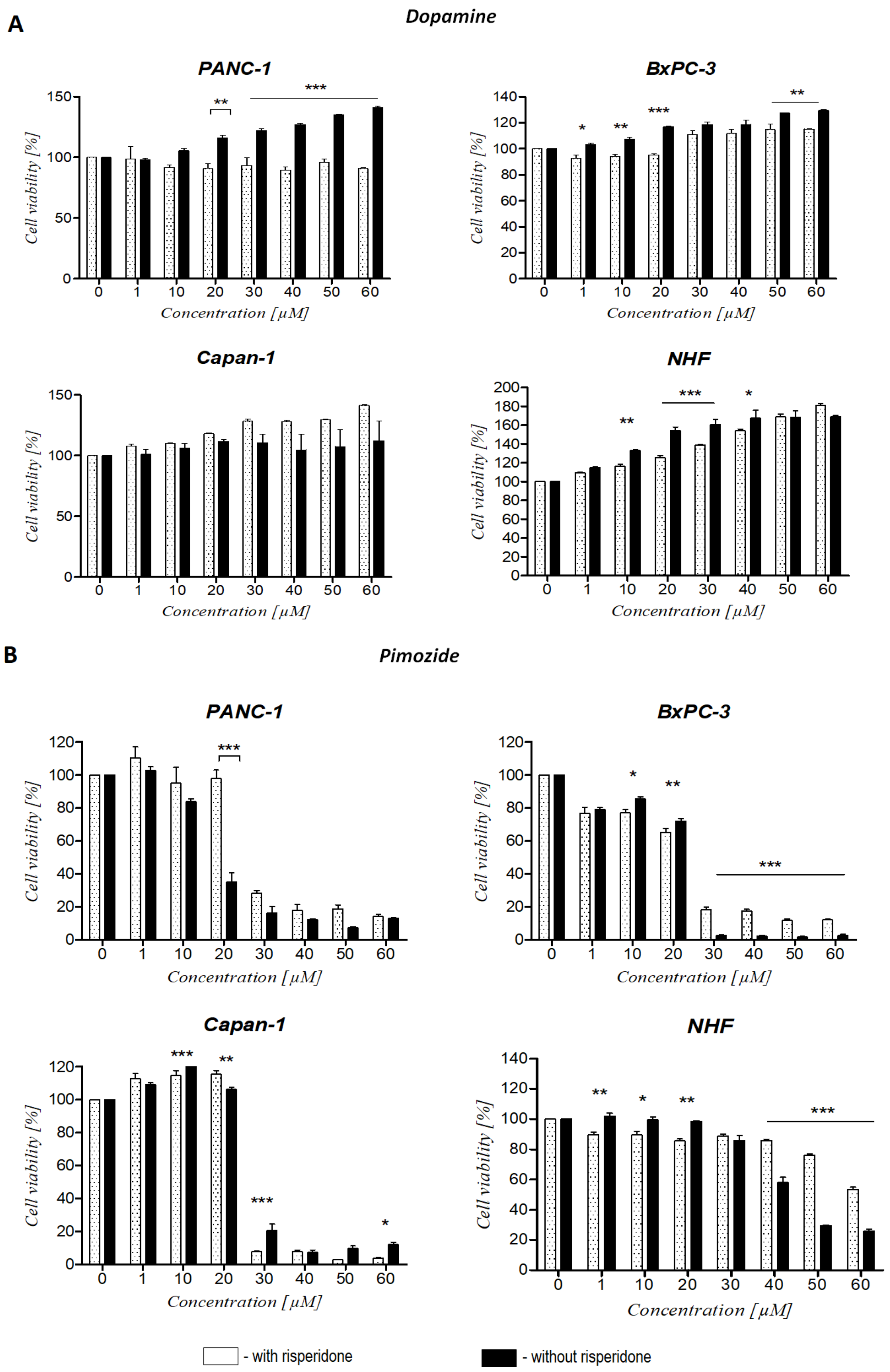

Figure S13. The comparison between the viability of risperidone-pretreated cells exposed to dopamine (A) or pimozide (B) and cells treated with dopamine or pimozide alone for 24h. Statistical analysis was performed using two-way ANOVA followed by Bonferroni post-tests. Results were considered statistically significant when: $^*P < 0.05$; $^{**}P < 0.01$; $^{***}P < 0.005$

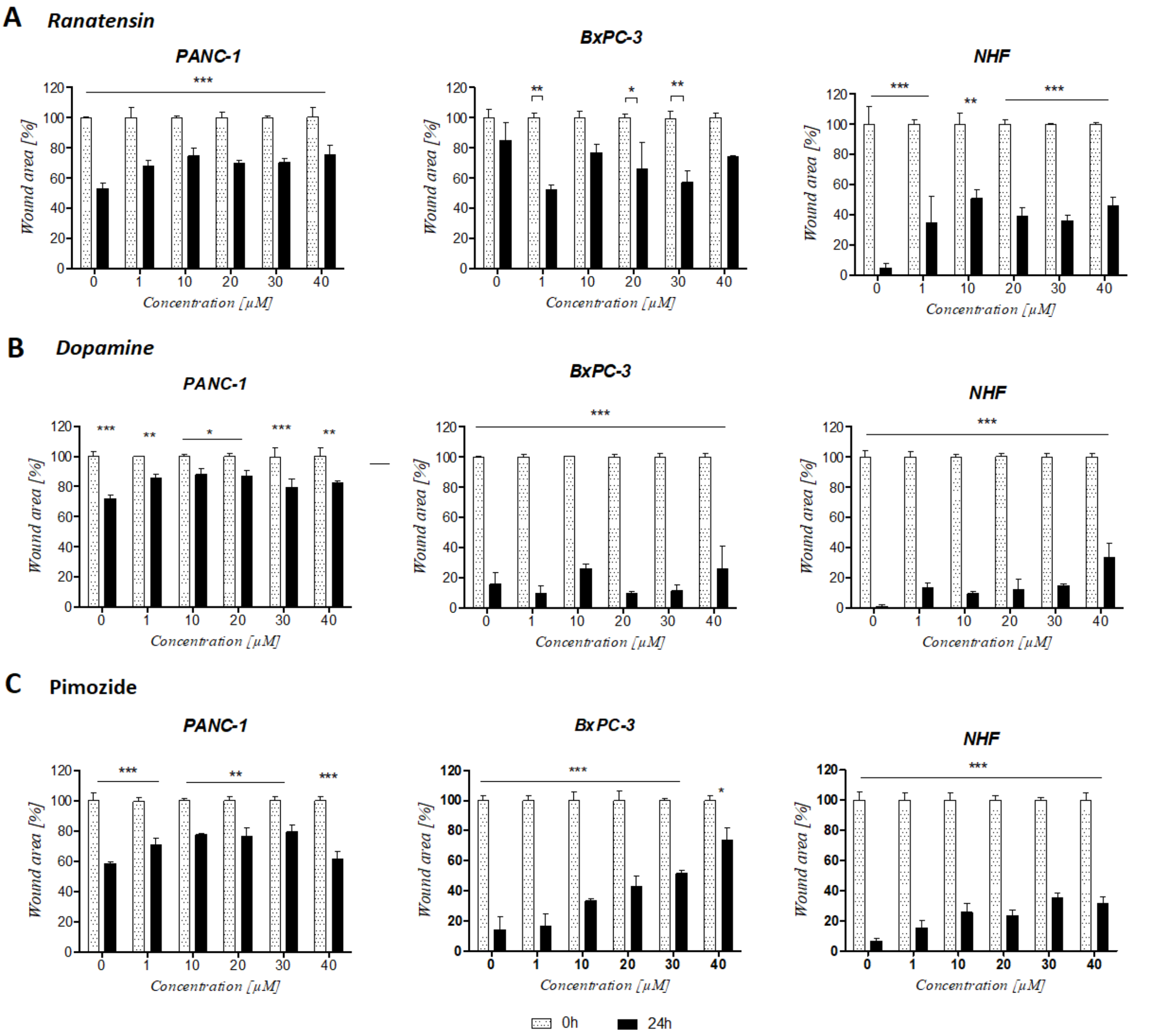

Figure S14. The effect of compounds on the cell migration of risperidone-pretreated cells. Representative phase-contrast microscope images showing the area covered by the cells at 0 and 24h after wounding, and presented in Figure S10. Original magnification 10x. Wound area (%) was determined by the rate of cells moving towards the scratched area upon time using ImageJ ™ software. Differences in wound area of cells treated with increasing concentrations of RAN at 0 and 24h were analyzed. Statistical analysis was performed using two-way ANOVA followed by Bonferroni post-tests. Results were considered statistically significant when: *P < 0.05; **P < 0.01; ***P < 0.005.

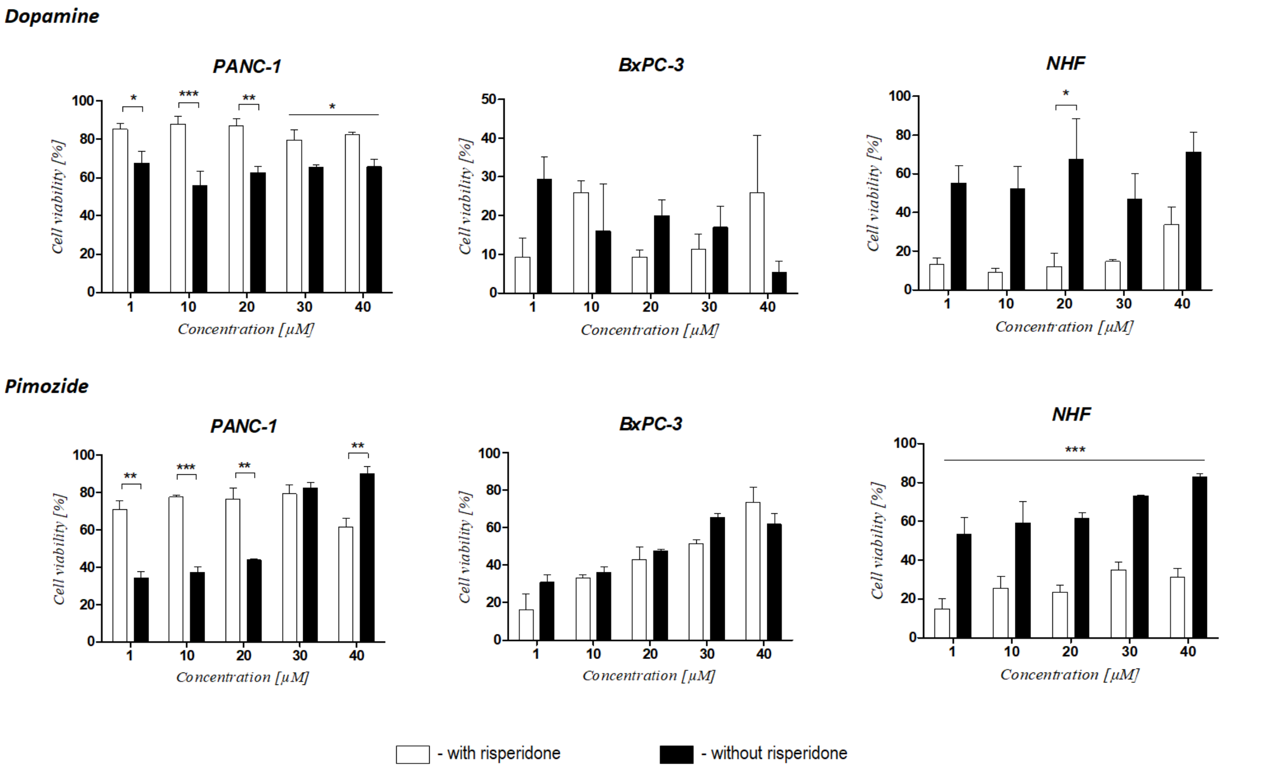

Figure S15. Comparison of the effect of dopamine and pimozide on the migration of risperidone-pretreated cells and cells treated with dopamine and pimozide alone after 24 h. Statistical analysis was performed using two-way ANOVA followed by Bonferroni post-tests. Results were considered statistically significant when: *P < 0.05; **P < 0.01; ***P < 0.005

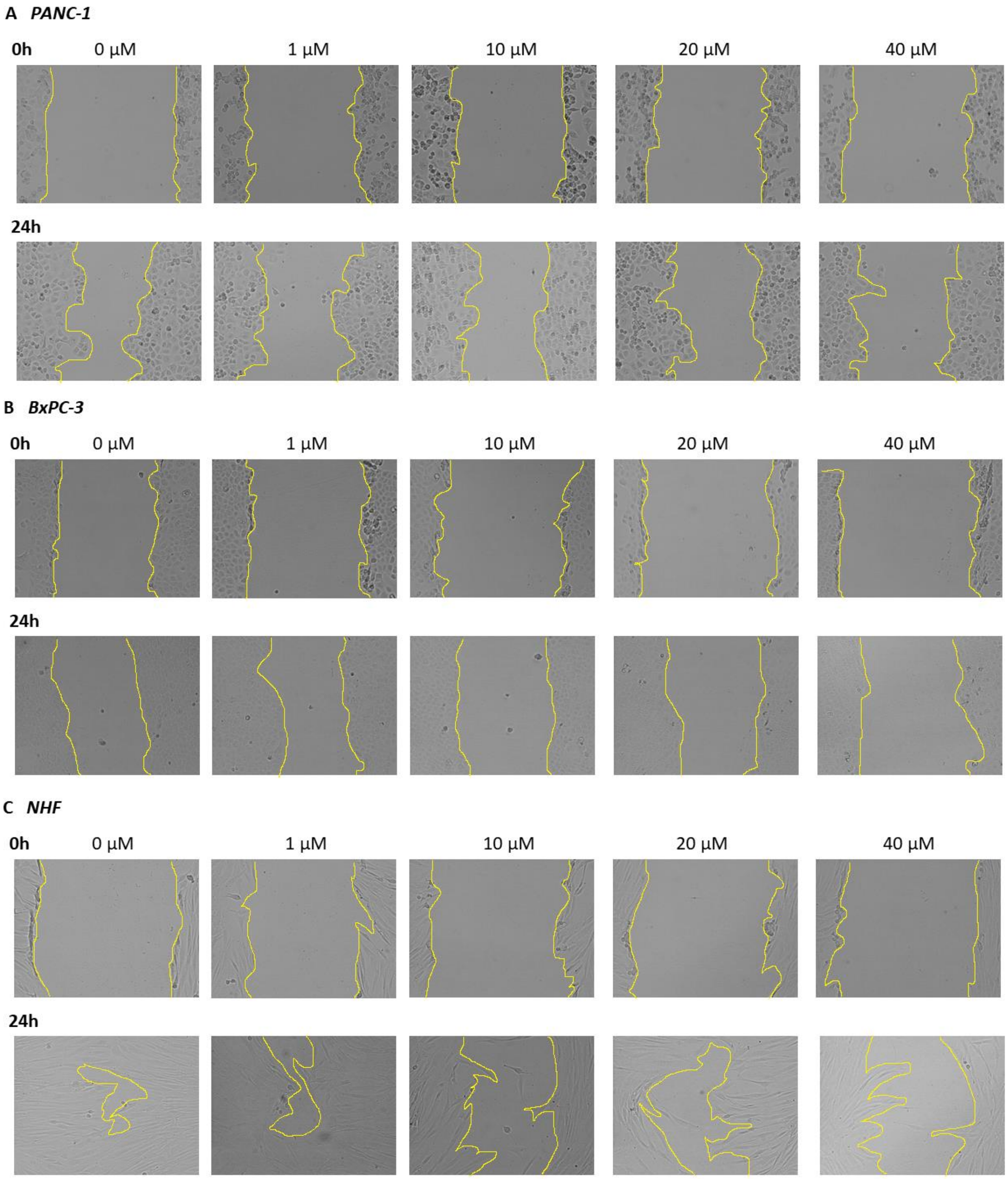

Figure S16. The effect of ranatensin on the cell migration of risperidone-pretreated cells. Wound-healing assays were performed at 0 and 24 on RAN treated and untreated PANC-1 (A), BxPC3 (B), and NHF (C) cells. Representative phase-contrast microscope images showing the area covered by the cells at 0 and 24h after wounding. Original magnification 10x. Wound area (%) was determined by the rate of cells moving towards the scratched area upon time using ImageJ ™ software, and presented in Figure S10.

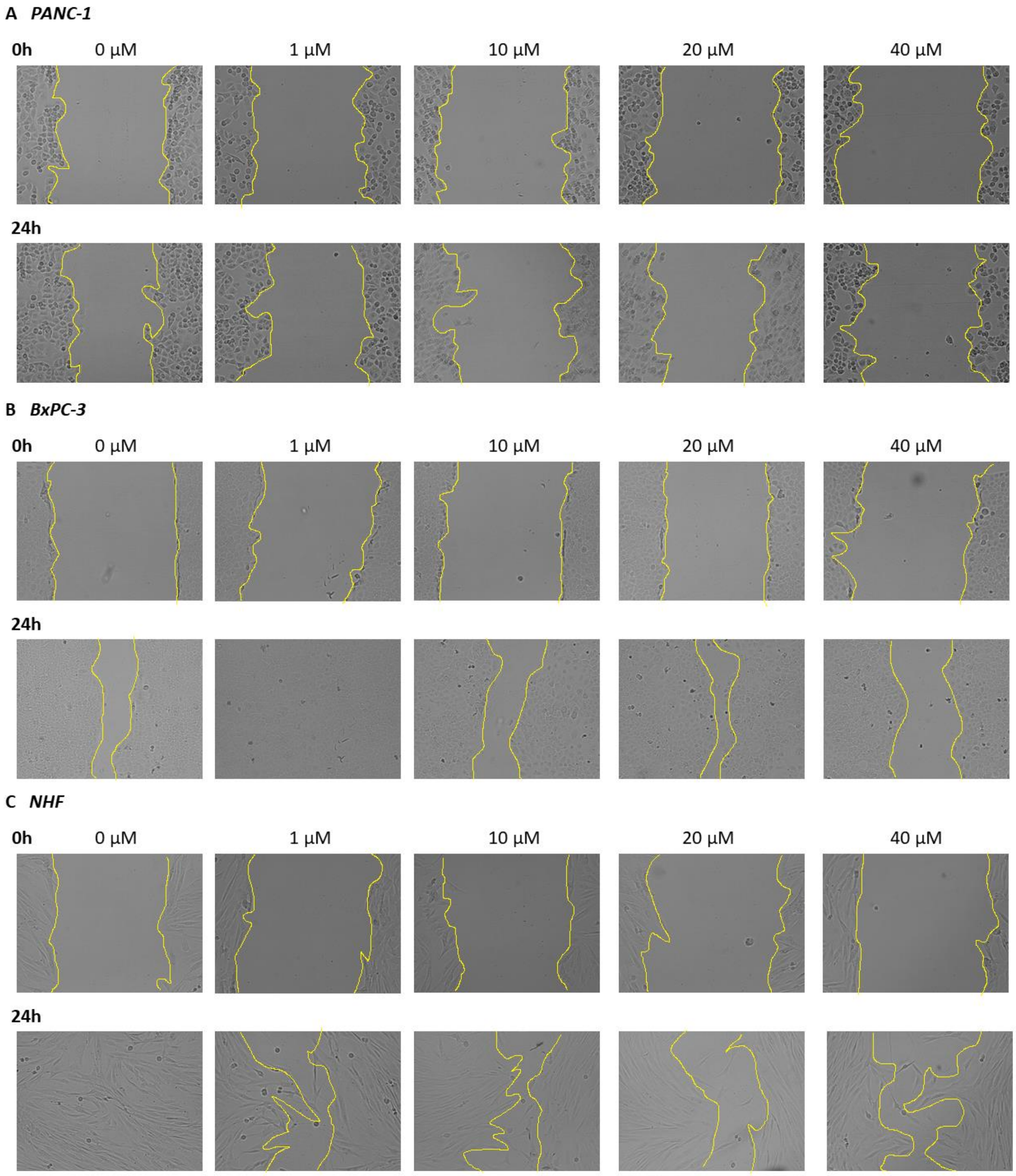

Figure S17. The effect of dopamine on the cell migration of risperidone-pretreated cells. Wound-healing assays were performed at 0 and 24 on dopamine-treated and untreated PANC-1 (A), BxPC3 (B), and NHF (C) cells. Representative phase-contrast microscope images showing the area covered by the cells at 0 and 24h after wounding. Original magnification 10x. Wound area (%) was determined by the rate of cells moving towards the scratched area upon time using ImageJ ™ software, and presented in Figure S10.

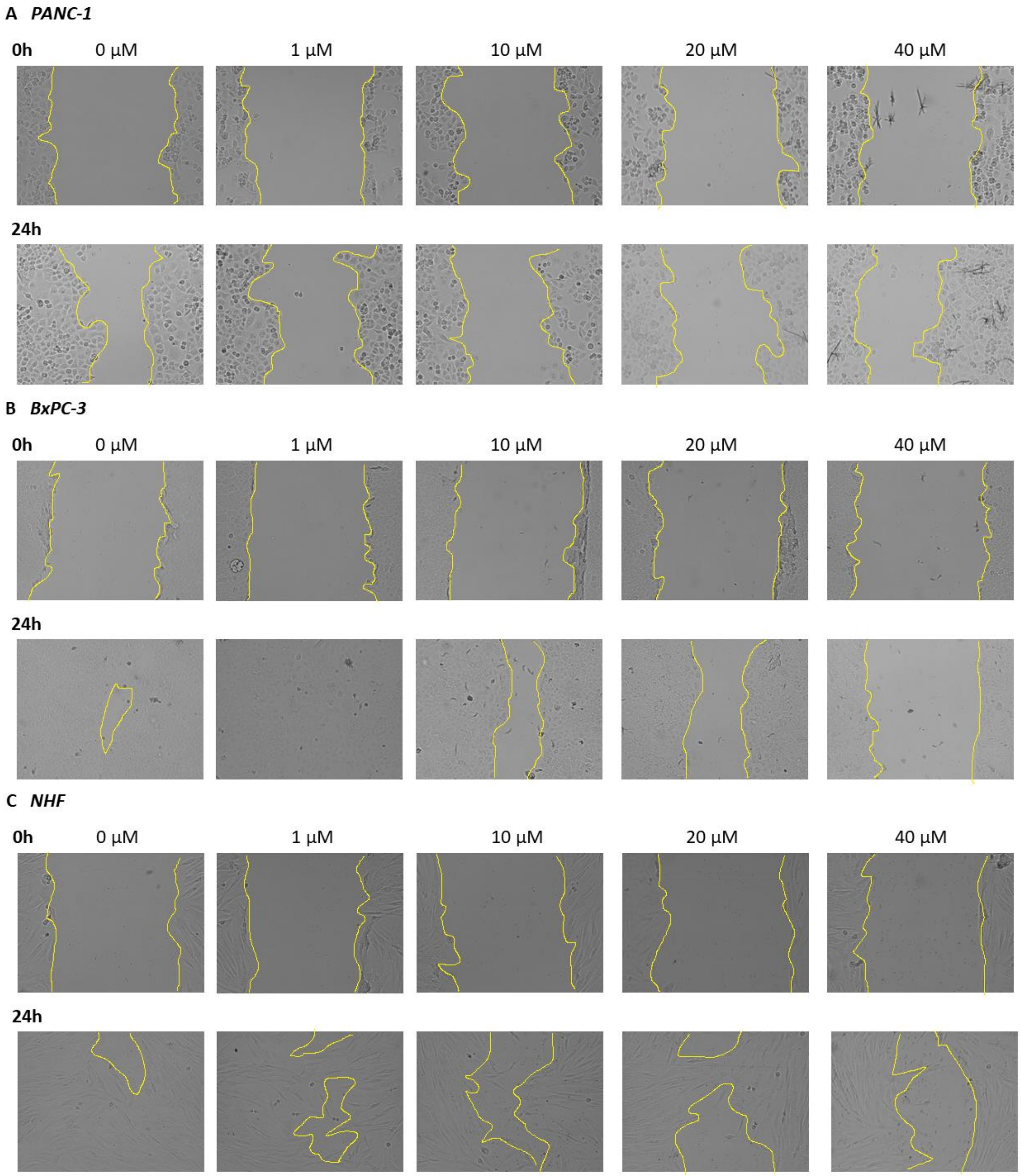

Figure S18. The effect of pimozide on the cell migration of risperidone-pretreated cells. Wound-healing assays were performed at 0 and 24 on pimozide-treated and untreated PANC-1 (A), BxPC3 (B), and NHF (C) cells. Representative phase-contrast microscope images showing the area covered by the cells at 0 and 24h after wounding. Original magnification 10x. Wound area (%) was determined by the rate of cells moving towards the scratched area upon time using ImageJ ™ software, and presented in Figure S10.

Table S1. IC_{50} values (μM) of compounds after 48 h of treatment.

	IC_{50} (μM)				
**Cell line**	**Ranatensin**	**Dopamine**	**Pimozide**	**Risperidone**	**Gemcitabine**
PANC-1	>60	>60	16.97 ± 1.08	>60	>60
BxPC-3	>60	>60	16.05 ± 0.94	>60	0.6426 ± 0.05
Capan-1	>60	>60	20.71 ± 1.3	>60	>60
NHF	>60	>60	28.71 ± 3.82	>60	>60

Table S2. IC_{50} values of DRD2 -blocked cells treated with pimozide for 24 h (μM)

	IC_{50} (μM)			
**Cell line**	**PANC-1**	**BxPC-3**	**Capan-1**	**NHF**
Pimozide	25.70 ± 1.65	22.41 ± 2.02	27.85 ± 2.81	>60
